# Diphenhydramine increases the therapeutic window for platinum drugs by simultaneously sensitizing tumor cells and protecting normal cells

**DOI:** 10.1002/1878-0261.12648

**Published:** 2020-03-10

**Authors:** Margarita Melnikova, Ulrike Sophie Wauer, Diana Mendus, Ralf Axel Hilger, Trudy G. Oliver, Kim Mercer, Björn Oliver Gohlke, Kati Erdmann, Dieter Niederacher, Hans Neubauer, Paul Buderath, Pauline Wimberger, Jan Dominik Kuhlmann, Jürgen Thomale

**Affiliations:** ^1^ Institute of Cell Biology (Cancer Research) University of Duisburg‐Essen Medical School Germany; ^2^ Department of Gynecology and Obstetrics Medical Faculty and University Hospital Carl Gustav Carus Technische Universität Dresden Germany; ^3^ National Center for Tumor Diseases (NCT), Dresden, Germany; German Cancer Research Center (DKFZ), Heidelberg, Germany; Faculty of Medicine and University Hospital Carl Gustav Carus, Technische Universität Dresden, Dresden, Germany; Helmholtz‐Zentrum Dresden ‐ Rossendorf (HZDR) Dresden Germany; ^4^ German Cancer Consortium (DKTK) Dresden and German Cancer Research Center (DKFZ) Heidelberg Germany; ^5^ West German Cancer Center University Hospital Essen Germany; ^6^ Massachusetts Institute of Technology Cambridge MA USA; ^7^ Structural Bioinformatics Group Institute for Physiology Charité – University Medicine Berlin Germany; ^8^ Department of Urology Medical Faculty and University Hospital Carl Gustav Carus Technische Universität Dresden Germany; ^9^ Department of Obstetrics and Gynecology University Hospital and Medical Faculty of the Heinrich Heine University Düsseldorf Germany; ^10^ Department of Gynecology and Obstetrics University Hospital Essen Germany; ^11^ Present address: Genentech, Inc. South San Francisco CA USA; ^12^ Present address: Department of Oncological Sciences University of Utah Salt Lake City UT USA

**Keywords:** cisplatin, drug import/export, platinum DNA adducts, MRP transporter family, ovarian cancer, oto-/ nephro-/ neurotoxicity

## Abstract

Platinum‐based compounds remain a well‐established chemotherapy for cancer treatment despite their adverse effects which substantially restrict the therapeutic windows of the drugs. Both the cell type‐specific toxicity and the clinical responsiveness of tumors have been associated with mechanisms that alter drug entry and export. We sought to identify pharmacological agents that promote cisplatin (CP) efficacy by augmenting the levels of drug‐induced DNA lesions in malignant cells and simultaneously protecting normal tissues from accumulating such damage and from functional loss. Formation and persistence of platination products in the DNA of individual nuclei were measured in drug‐exposed cell lines, in primary human tumor cells and in tissue sections using an immunocytochemical method. Using a mouse model of CP‐induced toxicity, the antihistaminic drug diphenhydramine (DIPH) and two methylated derivatives decreased DNA platination in normal tissues and also ameliorated nephrotoxicity, ototoxicity, and neurotoxicity. In addition, DIPH sensitized multiple cancer cell types, particularly ovarian cancer cells, to CP by increasing intracellular uptake, DNA platination, and/or apoptosis in cell lines and in patient‐derived primary tumor cells. Mechanistically, DIPH diminished transport capacity of CP efflux pumps MRP2, MRP3, and MRP5 particularly in its C2+C6 bimethylated form. Overall, we demonstrate that DIPH reduces side effects of platinum‐based chemotherapy and simultaneously inhibits key mechanisms of platinum resistance. We propose that measuring DNA platination after *ex vivo* exposure may predict the responsiveness of individual tumors to DIPH‐like modulators.

## Graphical abstract

The efficacy of platinum‐based cancer chemotherapies is hampered by both severe tissue‐specific toxicities and drug resistance of tumor cells. We report that the antihistaminic drug diphenhydramine (DIPH) and two methylated derivatives ameliorate cisplatin (CP)‐induced nephrotoxicity and ototoxicity in mice by blocking CP import into critical cells and simultaneously sensitize a variety of human tumor cells to CP by blocking CP export.

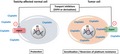

**Abbreviations**
AFU: arbitrary fluorescence units
BERA: brainstem‐evoked response audiometry
CDCF: 5(6)‐Carboxy‐2′,7′‐dichlorofluorescein
CP: cisplatin
DIPH: diphenhydramine
DRG: dorsal root ganglia
ICA: immuno‐cytological assay
ICP‐MS: inductively coupled plasma mass spectrometry
me_2_‐DIPH: 2‐(2.6‐dimethyl phenyl)‐phenyl‐metoxy)‐*N*,*N*‐dimethylethanamine
me‐DIPH: *N*,*N*‐dimethyl‐2‐(2‐methyl‐benzhydryl oxy)‐ethylamine
NER: nucleotide excision repair

## 1 Introduction

Platinum‐based drugs, especially cisplatin (CP) and carboplatin, have great utility in treating solid malignancies, including ovarian, cervical, testicular, and lung cancer (Galanski, 2006). The antineoplastic effect of platinum drugs derives from using various molecular mechanisms, including the generation of DNA platination products, such as DNA inter‐ and intrastrand adducts. When unrepaired, such lesions can trigger multiple cellular responses like mitochondrial apoptosis (Cohen and Lippard, 2001; Eastman, 1987; Galluzzi *et al.*, 2012; Jamieson and Lippard, 1999). Despite initial beneficial responses to platinum‐based chemotherapy in many patients, its long‐term success remains limited by intrinsic or acquired drug resistance and selective toxicity in normal cells, which prohibits dose escalation. Clinical responsiveness to platinum‐based chemotherapy varies widely between tumor types with moderate response rates for lung and colorectal cancer and very high response rates for seminoma. In ovarian cancer, approximately 80% of patients initially respond to platinum plus paclitaxel‐based chemotherapy, but intrinsic or acquired resistance typically develops over time (Eltabbakh and Awtrey, 2001; Galluzzi *et al.*, 2012). Mechanisms underlying platinum resistance utilize various routes like altering transmembrane trafficking (import or export) of platinum into tumor cells (pretarget resistance), increasing the cellular repair capacity of platinum DNA lesions (on‐target resistance), or activating lateral signaling pathways that bypass apoptosis (post‐target resistance) (Galluzzi *et al.*, 2012). As one example, pretarget resistance, for example, arises when tumor cells increase drug export via activating drug efflux pumps, such as ATP binding cassette subfamily C member 2 (ABBC2, also known as MRP2; Materna *et al.*, 2005).

Cisplatin therapy is often hampered by severe side effects in normal tissues like nephrotoxicity and ototoxicity (Karasawa and Steyger, 2015; Kelland, 2007), which limit its clinical benefit. To alleviate this problem, second‐generation platinum analogues, such as carboplatin or oxaliplatin, were designed for clinical use. Compared to cisplatin, carboplatin induces the same pattern of DNA adducts, displays less severe nephrotoxicity and ototoxicity and a lower molar efficacy, but increased hematotoxicity (Knox *et al.*, 1986). Specific assays for visualizing CP adducts (Melnikova and Thomale, 2018) can identify particular cell types that accumulate extremely high levels of DNA adducts (Dzagnidze *et al.*, 2007; Liedert *et al.*, 2006; Thomas *et al.*, 2006). Animal models showed that CP‐mediated toxicity correlates with an excessive formation of platinum adducts in the DNA of critical target cells of the kidney and inner ear. The instrumentation of those target cells with a complex repertoire of various ion transporters (Ciarimboli, 2014; George *et al.*, 2017; Ji *et al.*, 2013) suggests that aberrant drug import is a major risk factor for CP‐induced functional loss and structural damage. Several approaches exist to transiently block specific membrane importers to diminish typical side effects of CP (Huang *et al.*, 2017; Lanvers‐Kaminsky *et al.*, 2017).

These approaches can rely on antidromic and aberrant cellular import or export of the drug. So, modulating physiological or aberrantly expressed membrane transporters that drive these differential drug transport routes represents a key target to control toxicity and resistance simultaneously. Here, we identify the antihistaminic small‐molecule diphenhydramine (DIPH) and its methylated derivatives as agents that can protect against CP‐induced ototoxicity, nephrotoxicity, and neurotoxicity in mice and simultaneously sensitize tumor cells to platinum‐induced cell death.

## 2 Methods

### 2.1 Study approval

The study was approved by the Local Ethics Committee and performed according to good clinical practice guidelines, national laws, and the Declaration of Helsinki (TU Dresden: EK 74032013, EK 44022018; University Hospital of Essen: 17‐7859‐BO). Written consent was obtained from all patients. All animal experiments have been approved by the state animal welfare board (IFZ‐University Hospital of Essen, reference numbers: AZ 9.93.2.10.34.07.072, AZ 8.87‐50.10.34.08.251, AZ 50.05‐230‐4‐1/01; CAC Protocol MIT, Cambridge, MA, USA, reference number: # 0705 046 08).

### 2.2 Cell culture

Detailed information on all cell lines used in this study is provided in Table S1. All cell lines were maintained in a humidified incubator with 5% CO_2_ at 37 °C. All experiments were performed in mycoplasma‐free cells. All human cell lines have been authenticated using STR profiling within the last three years.

### 2.3 Primary patient‐derived ovarian cancer cells

Freshly resected human tumor tissue and/or ascites were available from the Department of Gynecology and Obstetrics (Technische Universität Dresden, Germany and University Hospital Essen, Germany). Tumor tissue was sliced into small pieces and was incubated with collagenase (1 mg·mL^−1^) for 1 h at 37 °C. Cells were separated from tissue residues by a strainer (70 µm). Mononuclear single‐cell fractions were isolated from ascites or from collagenase‐treated tissue using gradient centrifugation (Ficoll‐Paque Plus; GE Healthcare, Uppsala, Sweden) and transferred to RPMI‐1640 culture medium.

### 2.4 Immuno‐cytological assay for Pt‐(GpG) adducts in DNA

Cells in culture were exposed to CP for 3 or 4 h and (in case of kinetics studies) were maintained in drug‐free medium for up to 48 h. Cell aliquots taken at various time points were washed in PBS, resuspended, and placed onto microscopic adhesion slides (Superfrost Plus Gold Adhesion Slides; Thermo Fisher Scientific, Waltham, MA, USA). Immunostaining, visualization, and quantification of the DNA platination product Pt‐(GpG) in the nuclei of individual cells were performed as described (Melnikova and Thomale, 2018). Briefly, cells were fixed in methanol (−20 °C), denatured by alkaline treatment (60% 70 mm NaOH/140 mm NaCl; 40% methanol; 5 min; 0 °C), and digested successively with pepsin (400 µg·mL^−1^; 10 min; 37 °C) and proteinase K (400 µg·mL^−1^; 10 min; 37 °C). After blocking (5% skim milk in PBS), slides were stained with the Pt‐(GpG)‐specific rat antibody R‐C18 (20 ng·mL^−1^ in PBS/BSA; 12 h; 4 °C) (Liedert *et al.*, 2006) and visualized with Cy3‐labeled rabbit anti‐(rat IgG) antibody (#312‐165‐003; Dianova, Hamburg, Germany) for 1 h at 37 °C. Nuclear DNA was counterstained with DAPI (1 µg·mL^−1^ in PBS). DAPI‐ and Cy3‐derived signals were integrated and measured separately for individual cell nuclei using a microscope‐coupled digital image analysis system (Zeiss Axioplan; ACAS 6.0 Image Analysis System, Ahrens Electronics, Bargteheide, Germany). Antibody‐derived fluorescence signals were normalized to the corresponding DNA content of the same nucleus and expressed as arbitrary fluorescence units (AFU). Values were calculated as means of > 100 measured cells per sample; error bars represent 95% confidence intervals. The one‐way ANOVA test (*P* = 0.05) was used for the statistical analysis. Primary tumor cells were immunostained for the ovarian cancer antigen CA‐125 (1 : 1200; 60 min; RT; monoclonal mouse, clone OC125; ZYTOMED Systems, Berlin, Germany) prior to the immuno‐cytological assay (ICA). Images were documented, and the exact positions of positively stained cells on the slides were stored using a Tango positioning device (Märzhäuser, Wetzlar, Germany). Following ICA staining, CA‐125^+^ cells were revisited and adduct levels in the particular nuclei were determined.

### 2.5 ICP‐MS measurement of intracellular Pt levels

Inductively coupled plasma mass spectrometry (ICP‐MS) allows measurements of atomic Pt at trace concentrations of 5 ppt or lower. In order to measure the initial CP uptake, 10^6^ exposed cells were lysed by incubating the pellet with 1 mL 1% HNO_3_ for 24 h at 70 °C. Total platinum content was measured by ICP‐MS (820‐MS ICP Mass Spectrometer; Bruker Daltonics Inc., Billerica, MA, USA) and expressed as ng of Pt per 10^6^ cells. Values represent means of 20 scans per liquid sample with errors < 5%. Calibration curves were generated by using serial aqueous dilutions of standard reference material from the National Institute of Standards and Technology (NIST, Gaithersburg, MD, USA).

### 2.6 Mouse model of CP‐induced toxicity

C57Bl/6 mice were kept under specific pathogen‐free conditions at 12‐h light/dark cycle with free access to water and standard laboratory mouse food (‘10 H 10’; Eggersmann, Rinteln, Germany) *ad libitum*. Mice were weighed and checked for signs of drug toxicity before each application of CP. All experiments were performed when animals were aged 12–14 weeks. Male mice were from the IFZ in‐house breeding stock. All drugs were administrated at the given concentrations and time points by i.p. injection. If not specified, animals were sacrificed 16 h after last application or in case of repetitive treatments when weight loss was > 20% of initial values. All experiments have been approved by the state animal welfare board.

### 2.7 Tissue sections

Sacrificed mice were immediately dissected. Kidneys, dorsal root ganglia (DRG), and lungs were embedded in freezing medium (Tissue‐Tek, O.C.T., Sakura Finetek Europe, Alphen, the Netherlands) and shock‐frozen in liquid N_2_. Cochleae were dissected, fixed by intralabyrinthine perfusion with Carnoy's solution (3 h), decalcified in EDTA solution (10% w/v in PBS) for 3 days, and then placed in sucrose solutions (5% in PBS for 12 h and 15% in PBS for 5 h). Samples were embedded in Tissue‐Tek medium, frozen in liquid N_2_, and stored at −80 °C. Cryosections (8 μm) were prepared at −26 °C, placed onto adhesion slides (see above), air‐dried, and stored at −20 °C until further processing.

### 2.8 Electrophysiological examination of the hearing capability of mice: brainstem‐evoked response audiometry

Electrophysiological examinations were carried out with general anesthesia (xylazine, 20 mg·kg^−1^, plus ketamine, 100 mg·kg^−1^; i.p.) and controlled body temperature by placing the mice on a heated (37 °C) thermal blanket. Subdermal needle electrodes were inserted at vertex (active electrode), ventrolateral of left pinna (reference electrode), and ventrolateral of right pinna (ground electrode). For acoustic stimulus presentation, auditory brainstem response recording, and data management, a modified clinical potential system (NeuroScreen Plus, Toennies, Germany) was used. Repetitive clicks (substantial energy: 1–3 kHz; duration: 10 ms, rate: 39 s^−1^) were delivered through plastic tubes (diameter: 3 mm) to both ear canals (EAR Auditory Systems, Indianapolis, IN, USA). Brainstem‐evoked response audiometry (BERA) thresholds were determined by clicks beginning at 90 dB sound pressure level with decreasing intensity (10 dB‐steps) until the waves have lost reproducible morphology. The BERA thresholds were determined separately for each ear and by repetitive measurements. Mean values measured for drug‐treated mice were calculated as percentage of the mean hearing capability of the untreated control group. Following BERA, mice were sacrificed by cervical dislocation and both cochleae were removed for ICA.

### 2.9 Cell viability assay

Cell viability following drug treatment of human cancer cells *in vitro* was assessed using the CellTiter‐Blue^®^ Cell Viability Assay (Promega, Fitchburg, MA, USA) according to the manufacturer's instructions. Briefly, cancer cells were seeded at a density of 10 000 cells/well in a 96‐well plate. The cells were cultured in standard medium (Table S1) for 24 h to allow adherence. For short‐time CP treatment, cells were pretreated for 1 h with DIPH (and/or its derivatives) followed by DIPH+CP treatment for 4 h and viability readout after 48 h. For long‐term treatment, cells were pretreated for 4 h with DIPH (and/or its derivatives) followed by DIPH+CP treatment for 48 h. Viability readouts were performed with a fluorescence reader (Infinite M200; Tecan, Männedorf, Switzerland). For statistical analysis of viability data, two‐way ANOVA test was performed using prism 6.07 (GraphPad Software, San Diego, CA, USA).

### 2.10 Caspase 3/7 assay

In order to determine apoptosis‐associated caspase 3/7 kinetics following drug treatment, the Caspase‐Glo^®^ 3/7 Assay (Promega) was performed according to the manufacturer's instructions. Ovarian cancer cells were seeded at a density of 10 000 cells/well in a 96‐well plate. The cells were pretreated with DIPH (and/or its derivatives). After 4 h, CP was added and caspase 3/7 readout was performed after 48 h using a luminescence reader (Microplate Luminometer LB96 V; EG&G Berthold, Bad Wildbad, Germany). For statistical analysis, two‐way ANOVA test was used.

### 2.11 Reverse transcription and quantification by RT‐qPCR

Total RNA was extracted with the miRNeasy Mini Kit (Qiagen, Hilden, Germany) according to the manufacturer's instruction. Two hundred nanogram total RNA was reverse‐transcribed with the miScript II RT Kit (Qiagen). In order to relatively quantify MRP2, MRP3, and MRP5 mRNA in ovarian cancer cells, we utilized the following Primer Assays: Hs_ABCC2_1_SG QuantiTect Primer Assay, Hs_ABCC3_1_SG QuantiTect Primer Assay, Hs_ABCC3_va.1_SG QuantiTect Primer Assay, Hs_ABCC5_va.1_SG QuantiTect Primer Assay, and the Hs_GAPDH_vb.1_SG QuantiTect Primer Assay (all purchased from Qiagen). Quantitative RT‐qPCR was performed using the ABI 7500 FAST system (Applied Biosystems, Darmstadt, Germany).

### 2.12 Western blot analysis

Res2‐Igrov1 cells were grown to 80–90% subconfluency, trypsinized, and lysed in RIPA Lysis Buffer (Santa Cruz, Dallas, TX, USA). Subsequently, 20 µg whole cell lysate (per sample) was subjected to a NuPAGE 4–12% Bis‐Tris protein gel and transferred onto nitrocellulose (NC) membranes (Amersham™ Protran™ Premium 0.45 µm NC; GE Healthcare Life science, Chalfont St Giles, UK). Subsequently, (dissected) NC membranes were incubated with anti‐β‐actin antibody (AC‐74; Sigma‐Aldrich, Taufkirchen, Germany, 1 : 10^6^), anti‐cleaved PARP (Asp214) antibody (D64E10; Cell Signaling Technology, 1 : 250), or primary rabbit anti‐phospho‐γ‐H2AX antibody (#2577; Cell Signaling Technology, 1 : 250). Subsequently, membranes were incubated for detection with secondary antibodies, raised against rabbit and linked with HRP (#7074; Cell Signaling Technology) or raised against mouse and linked with HRP (order# 115‐035‐003; Dianova, Hamburg, Germany). Detection was performed with ECL Plus Western Blotting Detection reagent (GE Healthcare, order# RPN2232).

### 2.13 MRP vesicular transport assay

In order to analyze whether DIPH and its derivatives modulate MRP2, MRP3, or MRP5 transport capacity, we used the PREDIVEZ™ Vesicular Assay (SOLVO Biotechnology, Budaörs, Hungary) according to the manufacturer's instruction. This assay uses inverted MRP2, MRP3, or MRP5 vesicles from HEK293 cells and measures the import of the fluorescent MRP substrate dye 5(6)‐Carboxy‐2′,7′‐dichlorofluorescein (CDCF) in the presence or absence of DIPH or its derivatives. Fluorescence was detected with a fluorescence reader (Infinite M200; Tecan), and absolute quantification of transport rates was performed.

## 3 Results

### 3.1 DIPH decreases DNA platination in target cells of CP‐associated toxicity *in vivo*

**Figure 1:**
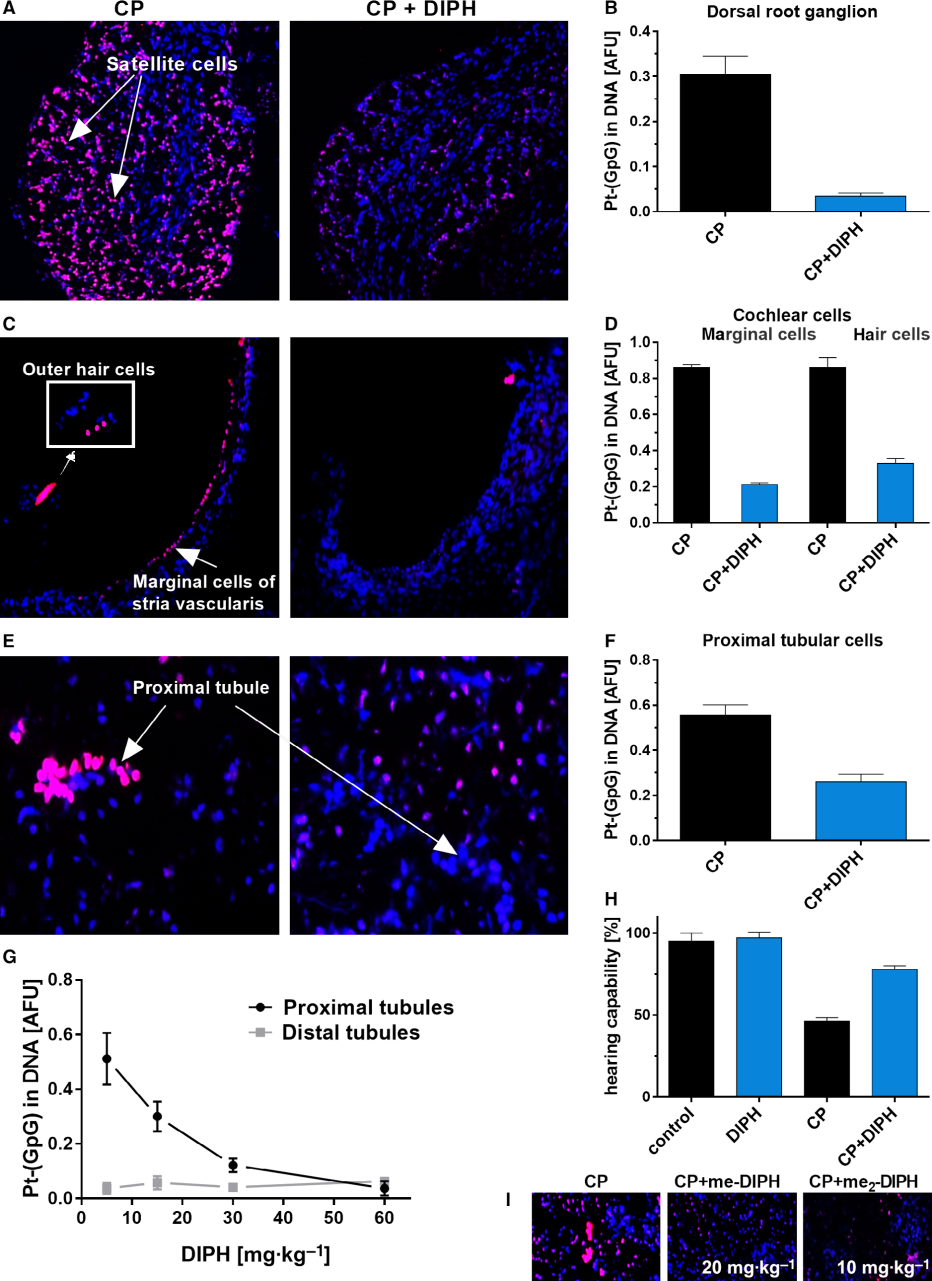
Diphenhydramine and its derivatives diminish excessive accumulation of Pt‐DNA damage in target cells of CP toxicity in mice. (A, C, E) Visualization and (B, D, F) measurement of Pt‐(GpG) adduct levels in the DNA of DRG (A), cochlea (C), and kidney cells (E) from mice 16 h after i.p. treatment with CP (10 mg·kg^−1^) alone (left) or in combination with DIPH (40 mg·kg^−1^, i.p., 1 h before CP) (right). Given the values for (B) DRG satellite cells, we also measured (D) marginal cells of the stria vascularis and outer hair cells and (F) renal proximal tubules cells. (G) DIPH dose‐dependent reduction of DNA platination in highly adducted proximal vs. distal tubule cells of CP‐treated mice. (H) Electrophysiological examination (BERA test) of hearing capability of untreated mice compared to mice treated with DIPH (twice weekly, 57 mg·kg^−1^; 2 weeks), CP (twice weekly: 5 mg·kg^−1^; for 2 weeks; cumulative dose: 20 mg·kg^−1^), or in combination with DIPH (57 mg·kg^−1^, 1 h before CP). Columns represent mean values (±SD) from five animals per treatment group and were calculated as percentage of the mean value of the control group. (I) *In vivo* effects of the derivatives me‐DIPH (20 mg·kg^−1^) and me_2_‐DIPH (10 mg·kg^−1^) on the Pt‐(GpG) adduct formation in the DNA of renal proximal tubule cells.

We screened small molecules that could attenuate the excessive accumulation of Pt‐DNA adducts in target tissues of CP‐induced toxicity in mice. We found the antihistaminic drug DIPH afforded the most protection when administered i.p. 1 h before CP injection (Fig. 1; Table S2). This pretreatment shielded mice from excessive DNA platination for specific cells in three predominantly affected tissue types, namely DRG satellite cells (Fig. 1A,B), stria vascularis marginal cells/outer hair cells of the cochlea (Fig. 1C,D), and epithelial cells of renal proximal tubules (Fig. 1E,F). The degree of DIPH protection was dose‐dependent. In the kidneys, DIPH gradually reduced high DNA platination levels in proximal tubular cells to levels occurring in less affected distal tubules or renal cortices (Fig. 1G). Cells typically showing little toxicity like peripheral lymphocytes or hepatocytes displayed no major alterations in their low DNA platination levels following concurrent DIPH administration (data not shown).

Next, we analyzed whether the DIPH‐mediated reduction of drug‐induced DNA damage in critical cells could improve the functioning of affected organs after CP administration. We treated mice repeatedly with CP alone (twice weekly: 5 mg·kg^−1^ for 2 weeks) or in combination with DIPH (1 h prior to CP administration). BERA in anesthetized mice after receiving 20 mg CP per kg body weight cumulatively indicated a 55% reduction in hearing capability in the CP‐only group compared to untreated controls. Injections of DIPH alone did not influence the audiometric data; however, the DIPH+CP group still showed significant protection from functional hearing loss with 86% remaining (Fig. 1H).

To investigate whether structural derivatives of DIPH can provide the same or greater protective capacity against CP‐associated toxicity, we tested two DIPH derivatives carrying one or two additional methyl groups in the ortho‐position at one phenyl residue (structural formula: Fig. S1). These compounds are referred to as *N*,*N*‐dimethyl‐2‐(2‐methyl‐benzhydryl oxy)‐ethylamine (me‐DIPH, also known as the approved drug orphenadrine) and 2‐(2.6‐dimethyl phenyl)‐phenyl‐metoxy)‐*N*,*N*‐dimethylethanamine (me_2_‐DIPH). The effective dose to reduce DNA platination of > 50% in renal tubules cells was 20 mg·kg^−1^ for me‐DIPH and 10 mg·kg^−1^ for me_2_‐DIPH when injected i.p. 1 h prior to CP in mice (Fig. 1I). When employing a small number of female instead of male C57Bl/6 mice, very similar results were obtained, indicating sex‐independent effects of DIPH. These findings indicate an enhanced protective effect by increasing the number of methyl groups at one phenyl ring. We only observed a transient, moderate sedation and decreased body temperature, but no major adverse effects using these DIPH compounds. Finally, we determined whether co‐application of me_2_‐DIPH also reduced systemic CP toxicity (Fig. S2). Male C57Bl/6 mice, repeatedly treated with a high dose of CP (10 mg·kg^−1^ weekly for 4 weeks), had a 100% survival rate when regularly pre‐injected with me_2_‐DIPH compared to 25% survival in the CP‐only group (*n* = 4 per group).

Together, these data demonstrate that DIPH and its structural derivatives (me‐DIPH and me_2_‐DIPH) strongly protect critical target cells from excessive DNA platination *in vivo* and attenuate CP‐associated nephrotoxicity, ototoxicity, and neurotoxicity.

### 3.2 DIPH increases intracellular CP uptake and DNA platination in tumor cells

**Figure 2:**
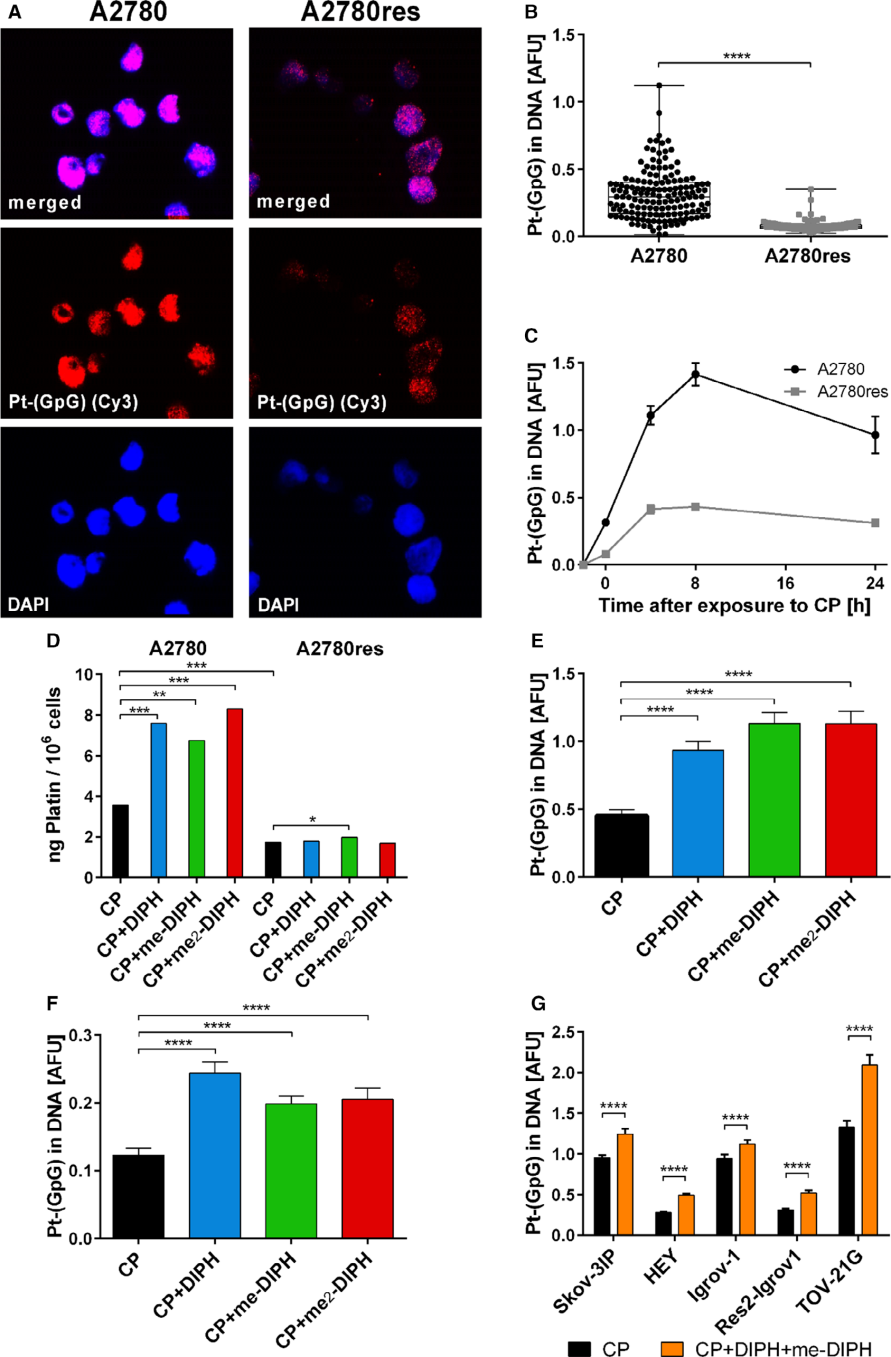
Intracellular CP level and DNA platination in CP‐exposed ovarian cancer cells increased following co‐application of DIPH and its methylated derivatives. (A) Imaging of Pt‐(GpG) adduct formation in the DNA of ovarian cancer cell lines A2780 (left) and the CP‐resistant variant A2780res (right) after exposure to CP (20 µg·mL^−1^ for 3 h). (B) Measurement of Pt adduct levels in the nuclear DNA of individual ovarian cancer cells after 3‐h CP exposure and (C) 24‐h kinetics of formation and repair of the adducts during postexposure cultivation in drug‐free medium. (D) Total intracellular platinum concentration in A2780 and A2780res cells exposed for 3 h to CP (20 µg·mL^−1^) alone or in combination with DIPH (40 µg·mL^−1^) or me‐DIPH (20 µg·mL^−1^) or me_2_‐DIPH (10 µg·mL^−1^) each 30 min prior to CP and throughout the exposure. Analysis was performed in cell lysates by ICP‐MS with replication errors < 5%. (E, F) Influence of DIPH and its derivatives on the Pt‐(GpG) adduct levels in CP‐exposed A2780 and A2780res cells (treatment as in D; note different *y*‐scales in E and F). (G) Modulation of CP‐induced DNA platination in five ovarian cancer cell lines by combined pretreatment with DIPH (40 µg·mL^−1^) and me‐DIPH (20 µg·mL^−1^) for 1 h following 3‐h treatment with CP (20 µg·mL^−1^), DIPH, and me‐DIPH. Data represent means of > 200 measured nuclei. Error bars indicate 95% confidence intervals. Statistical analysis used was one‐way ANOVA test (*P < 0.05; **P ≤ 0.01; ***P ≤ 0.001; ****P ≤ 0.0001).

Since DIPH decreases DNA adduct formation in physiological cells associated with CP toxicity, we sought to determine whether DIPH simultaneously reduces DNA platination in tumor cells, which would be counterproductive for the antineoplastic efficacy of CP. So, we used ovarian cancer as a clinically relevant model system. We exposed platinum‐sensitive A2780 ovarian cancer cells and their platinum‐resistant isogenic counterpart (A2780res) to CP for 3 h and then cultured cells for an additional 24 h in drug‐free medium. ICA revealed that DNA platination peaked 8 h after exposure in both cell lines, but was consistently about three times lower in A2780res cells compared to parental A2780 cells (*P* < 0.0001; Fig. 2A–C). We confirmed the diverse baseline levels of platinum DNA adducts in both cell lines for a broad range of CP doses (Fig. S3). These observations are consistent with CP resistance in A2780res cells, which suggest reduced formation of DNA adducts upon CP exposure as an underlying mechanism in CP resistance. Using mass spectrometry, we found about twofold lower total intracellular platinum levels after 3 h of CP exposure were in A2780res cells compared to A2780 parental cells (Fig. 2D). This assay measures not only the minor part (< 10%) of platinum DNA‐bound, but the total CP amount and its reaction products with other cell constituents. Therefore, CP resistance of A2780res largely arises through decreased import or increased export of CP rather than increasing DNA repair capacity or other post‐target mechanisms.

Next, we investigated whether DIPH pretreatment can attenuate intracellular CP uptake and DNA platination in A2780/A2780res cells compared to normal mouse cochlear, renal, and DRG cells. Surprisingly, we found that DIPH and its derivatives significantly increased levels of DNA platination in both cell lines accompanied by augmented total platinum concentrations in parental A2780 cells (Fig. 2D–F). We confirmed this increase in DNA platination in both cell lines at multiple CP concentrations (Fig. S3) and in five independent ovarian cancer cell lines (Fig. 2G). Further, DIPH also increased DNA platination in an additional panel of 14 cell lines from other tumor types, including neuroblastoma, lung cancer, and testicular cancer (Table S3).

Next, we determined the impact of DIPH with CP treatment on primary tumor cells in an immune‐competent setting *in vivo*. We employed an established mouse model of KRAS‐driven lung cancer (Oliver *et al.*, 2010). When tumor‐bearing mice were treated repeatedly with CP, adding DIPH to the treatment regimen did not decrease the Pt adduct burden in the DNA of lung tumor cells *in situ* nor the treatment efficacy (average tumor size per number of nodules; Fig. S4). The absence of DIPH‐mediated sensitization to CP for primary lung tumors in mice is consistent with our observation that DIPH and me‐DIPH did not affect CP‐induced DNA adduct levels in human A549 lung cancer cells, whereas me_2_‐DIPH caused a 50% increase in DNA platination (Table S3).

Collectively, we demonstrate that DIPH and its derivatives enhance intracellular CP accumulation and CP‐mediated DNA platination in cancer cells *in vitro* in strong contrast to its protective effect on critical target cells of CP toxicity.

### 3.3 DIPH sensitizes tumor cells to CP in cell line‐specific manner

Using a panel of 13 CP‐sensitive or CP‐resistant ovarian cancer cell lines and five platinum‐resistant cell lines from prostate or bladder cancer (Table S1), we systematically screened how DIPH impacts the cytotoxic effect of CP. To compensate for potentially diverging biological effects of DIPH and its highly similar structural derivatives, we performed this screening assay with a mixture of DIPH and me‐DIPH at concentrations which did not confer toxicity in the absence of CP (Fig. S5).

**Figure 3:**
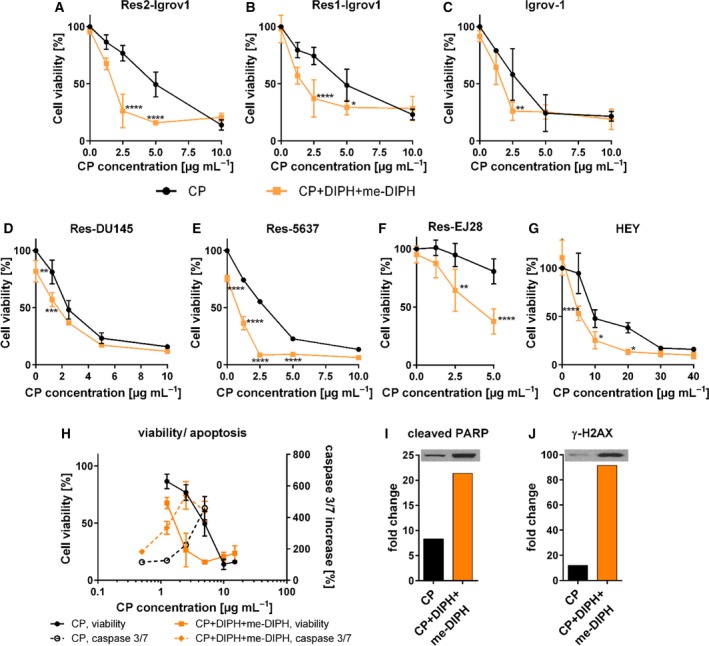
Diphenhydramine and its derivatives increase cytotoxic efficacy of CP in tumor cells. (A–E) Viability dose–response curves following CP treatment (black line) or pretreatment for 4 h with DIPH (40 µg·mL^−1^) and me‐DIPH (20 µg·mL^−1^) followed by treatment with CP, DIPH, and me‐DIPH for 48 h (orange line). (F) Viability dose–response curve following adapted treatment with CP or DIPH (20 µg·mL^−1^), me‐DIPH (10 µg·mL^−1^), and CP in Res‐EJ28 bladder cancer cells. (G) HEY ovarian cancer cells were pretreated for 1 h with DIPH (40 µg·mL^−1^) and me‐DIPH (20 µg·mL^−1^) followed by 4 h short‐time treatment with CP. Cell viability was measured after 48 h. (H) Viability dose–response curves (continuous line) and caspase 3/7 activity (dashed line) in platinum‐resistant Res2‐Igrov1 cells, following CP (black line) or CP with DIPH and me‐DIPH treatment (orange line). (I, J) Western blot analysis of cleaved PARP (89 kDa) and γ‐H2AX (15 kDa) in Res2‐Igrov1 cells following CP (black bars) or CP with DIPH and me‐DIPH (orange bars) treatment. Normalized percentages of cell viability and caspase 3/7 activity were averaged from three independent experiments. Data are reported as mean ± SD. For statistical analysis, two‐way ANOVA test was used (*P < 0.05; **P ≤ 0.01; ***P ≤ 0.001; ****P ≤ 0.0001).

DIPH+me‐DIPH did not inhibit the overall cytotoxic effect of CP regardless of treatment duration: (a) long‐term treatment approach (4 h pre‐incubation with DIPH+me‐DIPH, followed by 48‐h DIPH+me‐DIPH+CP treatment) or (b) short‐term treatment with a pulse exposure of escalated CP dosage (1 h pre‐incubation with DIPH+me‐DIPH, followed by 4‐h DIPH+me‐DIPH+CP treatment, Figs S6 and S7). Strikingly, DIPH+me‐DIPH sensitized cells to CP in 7/18 cell lines, in the majority platinum‐resistant (Fig. 3). This effect was most prominent in platinum‐resistant Res1‐Igrov1, Res2‐Igrov1, and isogenic Igrov‐1 platinum‐sensitive cells, in which DIPH+me‐DIPH alone did not alter cell viability but strongly increased CP‐mediated toxicity across the entire CP treatment range with high statistical significance (Fig. 3A–C). We reproduced platinum sensitization in Res2‐Igrov1 cells using carboplatin up to 150 µg·mL^−1^ (Fig. S8). We also observed comparable CP sensitization effects in platinum‐resistant Res‐EJ28, Res‐DU145, and Res‐5637 cells, albeit accompanied by residual additive toxicity conferred by DIPH+me‐DIPH alone (Fig. 3D–F). We found an additional CP‐sensitizing effect in HEY ovarian cancer cells only in the short‐term exposure regimen (Fig. 3G). We suggest this reflects the pharmacokinetic dosing *in vivo*.

Together, we report that in contrast to its protective effect against CP‐induced toxicity, DIPH (and particularly its derivatives) can act as platinum sensitizers in CP‐sensitive and CP‐resistant ovarian, bladder, and prostate cancer cells depending on the cellular context.

### 3.4 DIPH increases CP‐mediated DNA damage and apoptosis

For Res2‐Igrov1 cells, which showed the most consistent DIPH‐mediated CP sensitization effect, we observed that DIPH significantly increased CP‐mediated apoptosis as indicated by augmented caspase 3/7 induction and PARP cleavage (Fig. 3H,I). DIPH also significantly increased the formation of CP‐induced double‐strand breaks indicated by phosphorylated γH2AX (Fig. 3J). Interestingly, in cell lines that could not be sensitized by DIPH+me‐DIPH, like TOV‐21G cells, DIPH also increased DNA platination. However, this did not translate into an increased apoptotic caspase 3/7 response (Fig. S9).

Taken together, we report that DIPH and its derivatives do not attenuate the cytotoxic effect of CP in tumor cells, independent of treatment regime (long‐term vs. short‐term). Contrariwise, DIPH effectively sensitizes tumor cells to CP (and to carboplatin) in a cell line‐specific manner.

### 3.5 DIPH derivatives me‐DIPH and me_2_‐DIPH have superior capacity to sensitize for CP

Since we previously used a combination of DIPH+me‐DIPH, we dissociated the possible diverging effects of DIPH and derivatives on CP sensitization of tumor cells and added bimethylated me_2_‐DIPH to our experiments. me‐DIPH was slightly more effective in CP sensitization of Res2‐Igrov1 cells than DIPH. However, optimal sensitization still required a concentration of 80 µg·mL^−1^ for both drugs. Strikingly, while me_2_‐DIPH showed dose‐dependent toxicity at concentrations > 40 µg·mL^−1^ *in vitro*, a me_2_‐DIPH concentration of 20 µg·mL^−1^ was sufficient for effective sensitization of Res2‐Igrov1 cells to CP (Fig. S10).

We conclude that me_2_‐DIPH is about fourfold more efficient in CP sensitization of ovarian cancer cells than DIPH or me‐DIPH.

### 3.6 DIPH and derivatives increase DNA platination in primary ovarian cancer cells

**Figure 4:**
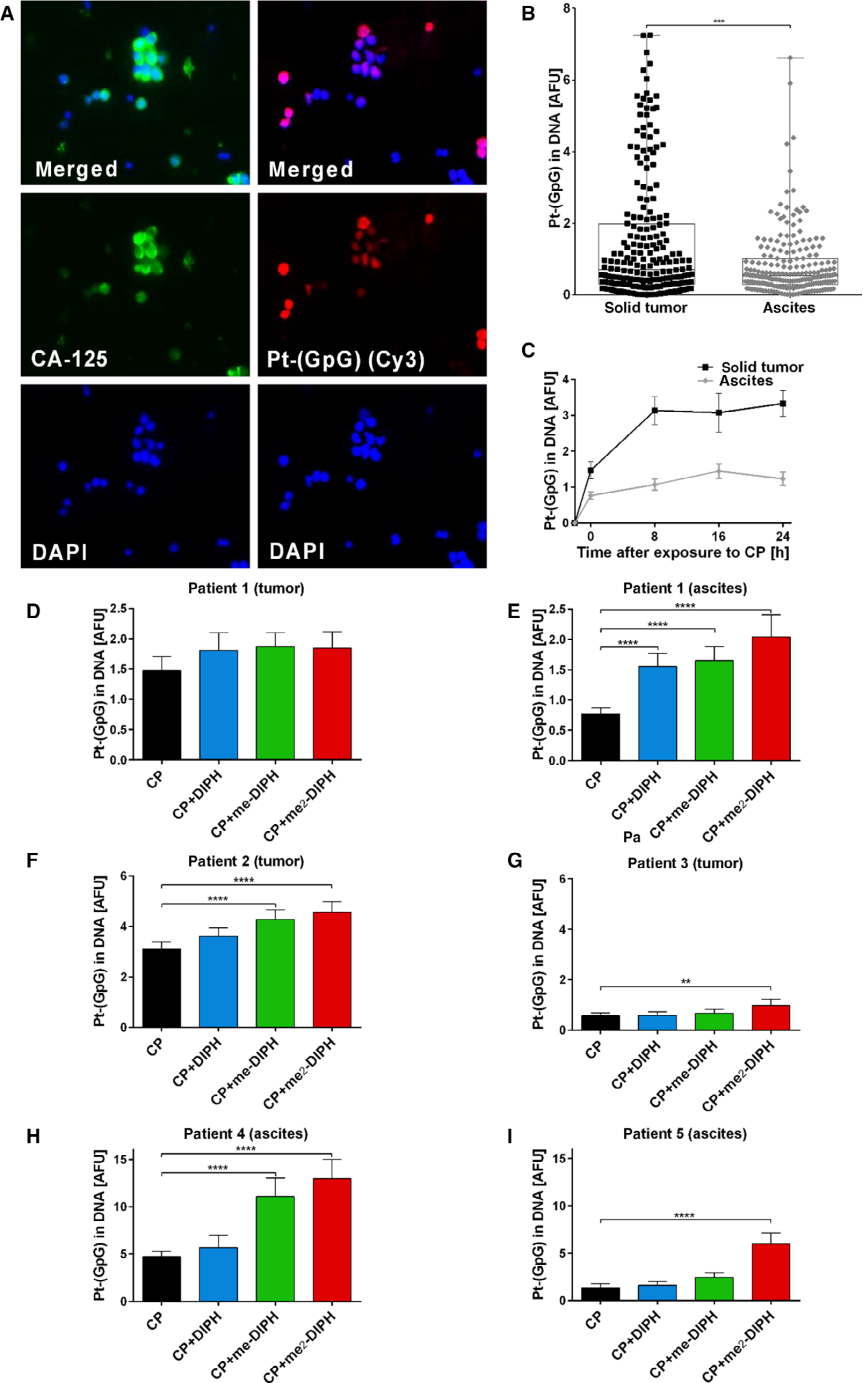
*Ex vivo* CP exposure of primary human ovarian cancer cells induced highly heterogeneous levels of DNA platination, which can increase intermittently with DIPH and its derivatives. (A) Localization of tumor cells by CA‐125 staining (green, left column), followed by visualization of CP‐induced Pt‐(GpG) DNA adducts (red, right column) in primary human ovarian cancer cells. Cells were dissociated from resected tumor tissue, exposed *ex vivo* to CP (20 µg·mL^−1^; 2 h), and analyzed by a two‐step procedure. (B) Adduct levels as measured in individual CA‐125‐positive tumor cells derived from solid tumor tissue or from ascites fluid of the same patient. (C) Formation and repair kinetics of Pt‐(GpG) adducts in ovarian cancer tumor cells (primary tissue, ascites, see B during cultivation for 24 h after CP exposure in drug‐free medium). (D, E) Modulation of Pt‐(GpG) adduct levels in the nuclear DNA of *ex vivo* CP‐exposed primary human ovarian cancer cells from tissue (D) or ascites (E) of the same patient (# 1, see B, C) by cotreatment with DIPH (40 g·mL^−1^) or me‐DIPH: (20 µg·mL^−1^) or me_2_‐DIPH (10 µg·mL^−1^; in each case 30 min before CP and throughout). (F–I) Impacts of DIPH and derivatives on the formation of Pt‐(GpG) adducts in the DNA of primary ovarian cancer cells from (F, G) fresh human tumor tissue or from (H, I) ascites of individual patients (#2–5). Error bars represent 95% confidence intervals. The one‐way ANOVA test was used for the statistical analysis (*P < 0.05; **P ≤ 0.01; ***P ≤ 0.001; ****P ≤ 0.0001). Data represent mean values of > 100 nuclei.

For clinical translation, we tested the effectiveness of DIPH in patient‐derived ovarian cancer cells from ascites fluid or primary ovarian cancer specimens. We established an experimental procedure enabling immediate *ex vivo* CP treatment (±DIPH) of freshly enriched primary tumor cells and subsequent quantification of DNA platination by immunostaining. CA‐125^+^ tumor cells from ovarian cancer tissue or ascites displayed very high intercellular variations in DNA platination ranging from 0.2 to > 6 AFU (Fig. 4A,B). However, the mean DNA platination level was significantly lower in cells from ascites fluid compared to primary cancer tissue simultaneously obtained from the same patient (*P* < 0.001; Fig. 4B). In both samples, DNA platination persisted without a significant clearance of Pt‐(GpG) intrastrand crosslinks from DNA within 24 h (Fig. 4C). We then tested whether primary ovarian cancer cells exposed to CP *ex vivo* respond to DIPH and its derivatives. Tissue‐derived tumor cells (Fig. 4D) displayed only marginally elevated DNA platination when pretreated with DIPH, me‐DIPH, or me_2_‐DIPH, whereas DNA platination in CA‐125^+^ tumor cells from ascites significantly increased up to 2.5‐fold (me_2_‐DIPH) with all three compounds (Fig. 4E). Analysis of additional independent patient samples confirmed this observation (Fig. 4F–I). Following the same exposure to CP (30 µg·mL^−1^; 2 h), cells from primary tumor or ascites displayed dramatically different mean adduct formation rates ranging from 0.5 to 3.5 AFU (Fig. 4F,G; *in situ*) and 1.2 to 4.8 AFU (Fig. 4H,I; ascites). However, all samples responded with minor to highly augmented DNA platination, when pre‐incubated with DIPH or its derivatives. Interestingly, me_2_‐DIPH induced the highest gain, despite having the lowest dose.

Based on these data, we conclude DIPH and its structural derivatives can augment DNA platination in patient‐derived primary ovarian cancer cells exposed to CP *ex vivo*.

### 3.7 DIPH and its derivatives decrease transport capacity of MRP drug efflux pumps

**Figure 5:**
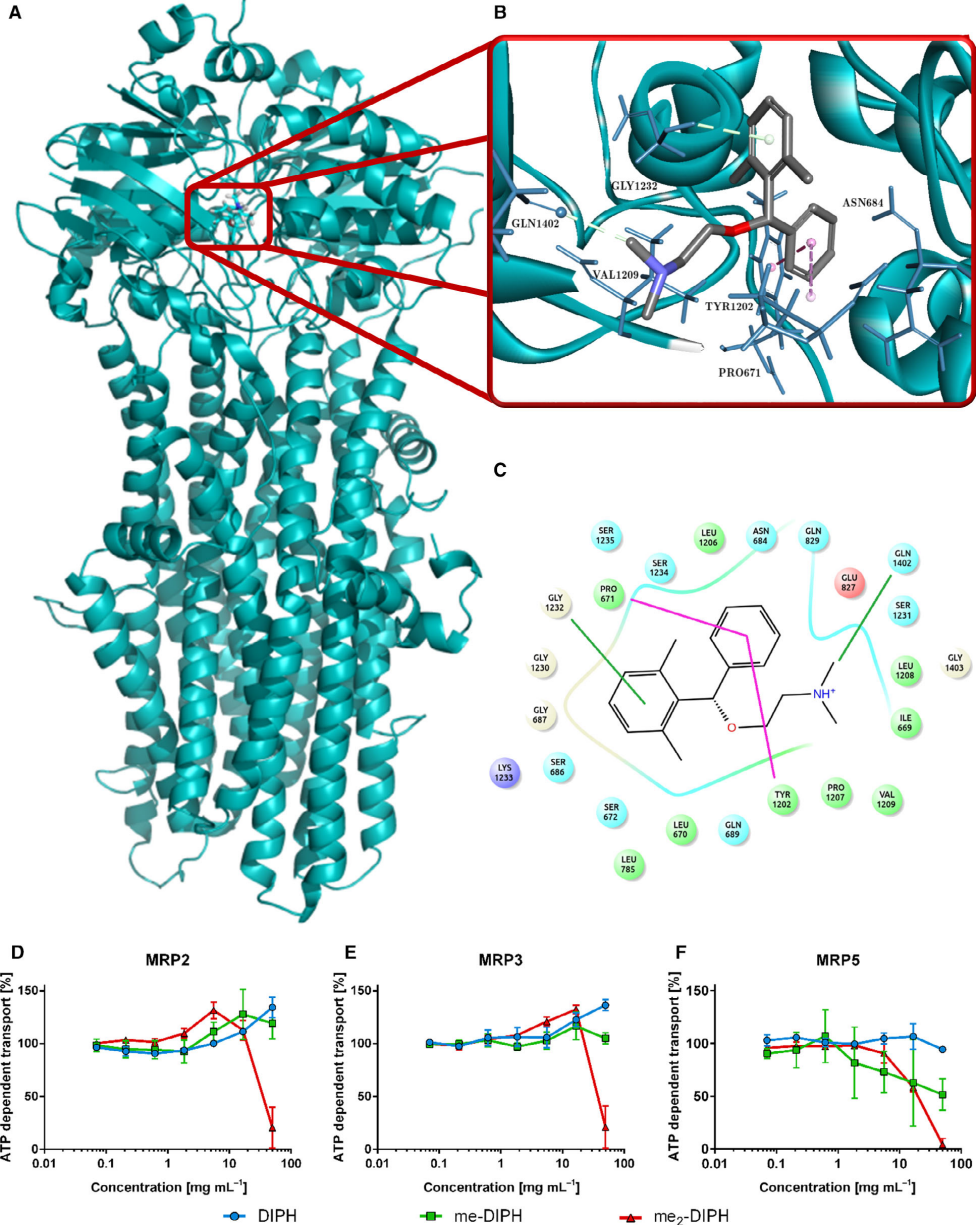
Diphenhydramine and derivatives decrease transport capacity of MRP drug efflux pumps. (A) Homology model of MRP5, based on the structural similar protein MRP1. Based on the high sequence similarity of those proteins, MRP5 was further investigated by docking studies. (B) The docking pose of me_2_‐DIPH in MRP5 protein with highlighted interactions (green: hydrogen bonds, purple: hydrophobic interactions). (C) 2D interaction diagram displays the docking pose of me_2_‐DIPH in MRP with 3‐letter code amino acids. (D–F) ATP‐dependent transport of a fluorescent substrate dye via (D) MRP2, (E) MRP3, and (F) MRP5 depending on increasing concentrations of DIPH (blue curve), me‐DIPH (green curve), or me_2_‐DIPH (red curve). Data were averaged from two independent experiments and are reported as mean ± SD.

Using a cheminformatics similarity approach, we sought to identify new molecular targets of DIPH. So, we screened for small molecules with high similarity to DIPH, with the assumption that DIPH could also target the known molecular targets of these similar compounds. We identified chlorprothixene and atomoxetine as sharing high three‐dimensional (but not two‐dimensional) similarities with DIPH (Fig. S11). Thus, we interrogated their known interaction partners. We predicted three drug efflux pumps of the ATP binding cassette subfamily (namely MRP2, MRP3, and MRP5) as potential molecular targets of DIPH, which are already associated with platinum resistance in ovarian cancer (Surowiak *et al.*, 2006). We subsequently confirmed this using *in silico* docking studies with MRP homology models (Fig. 5A–C). Based on the ratio from the docking scores to the respective ATP‐docking score, we assumed that DIPH, me‐DIPH, and me_2_‐DIPH have ascending affinity to the MRP drug efflux pumps, given their increasing number of methyl groups.

We then sought to validate these *in silico* findings. We confirmed Res2‐Igrov1 cells, which were most efficiently sensitized by DIPH, express *MRP2*, *MRP3,* and *MRP5* at mRNA level (Fig. S12). We then performed an ‘inside‐out’ vesicular uptake assay with inverted membrane vesicles to assess whether DIPH and its derivatives functionally interfere with the transport capacity of MRP drug efflux pumps (Fig. 5D–F). Influx rates for the fluorescent MRP substrate 5(6)‐Carboxy‐2′,7′‐dichlorofluorescein (CDCF) into membrane vesicles harboring either MRP2 or MRP3 or MRP5 remained mostly unchanged for all three transporters after DIPH incubation. In contrast, me‐DIPH mediated a gradual decline of MRP5 transport capacity, whereas MRP2 and MRP3 functions remained predominantly stable across the entire treatment range. Most strikingly, treatment of vesicles with me_2_‐DIPH > 10 mg·mL^−1^ caused robust and consistent inhibitory effects on all three MRP efflux pumps.

Taken together, we report that DIPH derivatives, particularly me_2_‐DIPH, bind to the drug efflux pumps MRP2, MRP3, and MRP5 and functionally impair their efflux transport capacity.

## 4 Discussion

Here, we identified the first‐generation antihistaminic drug, DIPH, and two derivatives that decrease DNA platination in key target cells of CP‐associated toxicity, which ameliorates nephrotoxicity, ototoxicity, and neurotoxicity in mice. However, DIPH did not attenuate the effects of CP in human tumor cell lines *in vitro* or in KRAS‐induced primary lung tumors in mice. Surprisingly, we found that DIPH efficiently sensitized tumor cells to CP in ovarian cancer in a cell line‐specific manner.

Cisplatin is a classical genotoxic drug, which causes DNA platination, followed by DNA damage response and mitochondrial apoptosis (Cohen and Lippard, 2001). Among the general principles of genotoxic agents, the cellular replication rate best correlates with CP‐associated cytotoxicity. So, fast replicating cells, such as tumor cells or specific normal cells, are usually more sensitive to CP than less replicative or postmitotic cells. Nevertheless, CP toxicity presents a spectrum of effects among postmitotic, terminally differentiated cells. The primary effect causes DNA platination that interferes with mRNA transcription and protein biosynthesis, leading to functional impairments or apoptosis/demise of those cells (Todd and Lippard, 2009).

We demonstrated that particular postmitotic normal cell types, such as proximal tubule cells of the kidney or stria marginal/outer hair cells of the cochlea, are severely affected by DNA platination after CP exposure. In contrast, there was little to no platination, in directly neighboring distal tubules or glomeruli. This result mimics the toxicity profile of CP in patients (Karasawa and Steyger, 2015). Our data strongly support the hypothesis that selectively strong DNA platination upon CP exposure in these target cells arises from an increased CP import and intracellular CP accumulation and not from other factors, such as differential DNA repair capacity (Dzagnidze *et al.*, 2007; Wang and Lippard, 2005). Overall, we speculate that the ability of normal or tumor cells to import (or export) CP underlies CP sensitivity. This ability is predefined by the individual expression profile of CP‐relevant membrane importers/exporters in each cell type.

We propose that DIPH, besides its known competitive antagonism of the histamine H1 receptor, concomitantly inhibits a spectrum of additional unidentified membrane transporter molecules as secondary targets. Our results suggest that these secondary DIPH targets partially regulate cellular CP import and/or export, which establishes a direct link between DIPH and modulating CP‐trafficking routes and cellular CP sensitivity. Based on cell‐specific expression of these secondary targets, DIPH and its derivatives exert differential effects on transmembrane pumping of CP in different cell types. In strongly CP‐affected tissue structures, such as epithelial cells of renal proximal tubules, DIPH may impair the drug import route by inhibiting CP‐relevant proteins to reduce total cellular Pt levels and DNA platination. We found no such effect in neighboring distal tubule cells (Fig. 1; Thomas *et al.*, 2006). This finding holds high clinical interest and may reposition DIPH as an auxiliary drug for platinum‐based chemotherapy by reducing CP‐associated side effects in patients. The potential side effects of DIPH are restricted to a mild sedative effect. No additional adverse effects of DIPH were observed in our experiments. The responsible membrane transporters mediating the effect of DIPH still remain unknown, and their identification is beyond the scope of the present study.

In tumor cells, which rely on a completely different spectrum of membrane transporters for CP trafficking, DIPH monotherapy exerted *in vitro* toxicity in a small subset of the tested tumor cell lines after continuous long‐term treatment (48 h). Tumor cells often overproduce acidic species due to metabolic reprogramming (known as the Warburg effect, Vander Heiden *et al.*, 2009), which must be extruded by voltage‐gated proton channels (Hv1) to maintain homeostasis. DIPH can trigger intracellular acidification by inhibiting Hv1 and its associated proton currents, which ultimately decreases intracellular pH and induces apoptosis (Asuaje *et al.*, 2018). We corrected for this effect by dosage adaption to reduce DIPH‐mediated single toxicity and unmask potential synergistic interactions with CP. Strikingly, we reported that DIPH does not decrease DNA platination as observed in the normal tissue, but rather significantly increases it (Fig. 2; Table S3) and sensitized it to CP in primarily platinum‐resistant ovarian cancer cells (Fig. 3). We speculate that CP sensitization is caused by a DIPH‐mediated modulation of CP trafficking likely through the intracellular drug level by inhibiting selective CP‐relevant membrane transporters. In line with this hypothesis, we experimentally defined functional correlates of this effect, as me_2_‐DIPH negatively modulates transport capacity of the drug efflux pumps MRP2, MRP3, and MRP5 (Fig. 5), which export CP and mediate platinum resistance, as demonstrated for ovarian cancer (Liedert *et al.*, 2003; Materna *et al.*, 2005; Surowiak *et al.*, 2006). Thus, we propose that DIPH sensitizes tumor cells to CP by inhibiting MRP‐mediated CP efflux.

The nonmethylated DIPH and me‐DIPH did not inhibit MRP activity in the vesicular assay. However, we hypothesize that all three drugs do inhibit MRP activity *in vivo*, due to the fact that (a) Res2‐Igrov1 cells express MRP2, MRP3, and MRP5 at mRNA level and were efficiently sensitized by DIPH and me‐DIPH; (b) the ratio from the docking scores to the respective ATP‐docking score strongly suggests that all three drugs have ascending affinity to the MRP drug efflux pumps (DIPH < me‐DIPH < me_2_‐DIPH); and (c) all three drugs have ascending CP‐sensitizing capacity *in vitro* (DIPH < me‐DIPH < me_2_‐DIPH). The vesicular assay creates an artificial experimental setting with MRP inside‐out vesicles from HEK293 cells and technically permits only a very short exposition time of MRP transporters with our DIPH drugs (< 20 min), which we exploited at supraphysiological concentrations. Therefore, we hypothesize that only me_2_‐DIPH with the highest affinity to MRP2, MRP3, and MRP5 confers an effect within the sensitivity range of the vesicular assay.

**Figure 6:**
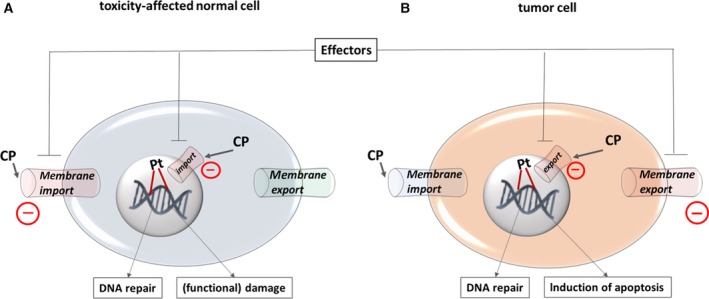
Pharmacological strategy of antidromic targeting CP transport mechanisms. Schematic shows how pharmacological effectors (such as DIPH or its derivatives) could influence antidromic CP transport mechanisms. (A) In toxicity‐affected normal cells (e.g., proximal tubule epithelial cells of the kidney), effectors reduce import of CP through the cytoplasmic (and/or nuclear) membrane, which decreases CP‐associated (functional) damage of these cells and ameliorates CP‐associated adverse effects. (B) In tumor cells, effectors simultaneously reduce export of CP through the cytoplasmic (and/or nuclear) membrane, which underlies key mechanisms of platinum resistance.

Platinum resistance is a complex phenomenon and can act on different functional layers (reviewed in Galluzzi *et al.*, 2012). In the A2780 *in vitro* model (Fig. 2) and primary ovarian cancer cells (Fig. 4), we hypothesized that their respective levels of DNA damage (and CP sensitivity) are predetermined by the differential import/export balance for the drug (pretarget resistance) rather than by modulating their DNA repair capacity (on‐target resistance). Our rationale for this prediction derives from: (a) Resistant A2780res cells demonstrated both reduced DNA adduct levels and decreased total intracellular platinum concentration and (b) DNA platination kinetics in A2780/A2780res and primary tumor cells exhibited only very moderate (cell lines) or almost no (primary ovarian cancer cells) repair of DNA platination over 24 h. Accelerated DNA repair, especially nucleotide excision repair (NER) in the context of platinum compounds, is well‐established and likely produces cellular drug resistance and clinical nonresponsiveness (Galluzzi *et al.*, 2012). However, NER is a complex mechanism involving the well‐coordinated expression and interaction of more than 20 proteins, so it is more likely that selective pressure by DNA‐damaging drugs preferably leads to altered expression of single elements, such as membrane transporters. Therefore, we conclude that DIPH reverts pretarget resistance in tumor cells (Fig. 6). We reproduced this effect with carboplatin (Fig. S7), which together with paclitaxel, comprises the first‐line treatment in ovarian cancer. Based on the frequently observed cross‐resistance between CP and carboplatin (Rixe *et al.*, 1996), we predict that the platinum‐sensitizing effect of DIPH is similar for CP and carboplatin. This mechanism could be exploited as an ovarian cancer treatment.

Bimethylated me_2_‐DIPH was approximately fourfold more efficient in CP sensitization than DIPH and me‐DIPH. Additional methyl groups at these aforementioned positions likely limit the degree of rotation freedom for the phenyl residues, which stabilizes conformation(s) of the molecule and increases its inhibitory effects on membrane transporters. We speculate this underlies why me_2_‐DIPH had the strongest CP‐protective effect in normal cells and the strongest MRP‐inhibitory capacity. However, some cell lines, most notably platinum‐resistant Skov‐3IP cells, were nonresponsive to DIPH‐mediated CP sensitization. In these tested lines, including Skov‐3IP, DIPH significantly enforced DNA platination but was not translated into an amplified apoptotic response. Since cultured cells may show different mechanisms of resistance, even at a subclonal level (McDermott *et al.*, 2014), we speculate that certain cells engage additional (e.g., post‐target) mechanisms and bypass excessive apoptosis by activating lateral resistance‐associated signaling pathways, such as the AKT/mTOR pathway (Peng *et al.*, 2010).

Various ‘protective’ pharmacological strategies for platinum‐based chemotherapy have been proposed (Baiceanu *et al.*, 2015; Ikemura *et al.*, 2017; Stojanovska *et al.*, 2017; Wensing and Ciarimboli, 2013). For example, amifostine, a cytoprotective adjuvant, can ameliorate neurotoxicity of platinum/taxane‐based chemotherapy of ovarian cancer (Hilpert *et al.*, 2005). However, we here define such an auxiliary drug from two perspectives: (a) its protective effect from CP‐associated side effects and (b) its neutral or favorable influence on the cytotoxic effect of CP on (platinum‐resistant) tumor cells. In both regards, DIPH showed clinically relevant effects, which we could reproduce in patient‐derived ovarian cancer cells. Our approach to measure DNA adduct levels in primary tumor cells exposed *ex vivo* to platinum drugs +/− inhibitors could serve as an assay predicting (a) drug sensitivity/drug resistance (high/low adduct levels) and (b) individual responsiveness to export inhibitors, like DIPH, introduced herein.

## 5 Conclusion

We demonstrate that DIPH, an FDA‐approved antihistaminic drug and antiemetic for cisplatin therapy (Tsavaris, 1992), antidromically modulates platinum transport routes in normal vs. tumor cells. Future studies will determine whether DIPH may aid platinum‐based chemotherapy as a drug‐repositioning approach. We envision that DIPH may improve chemotherapy tolerance, allow dosage escalation, and simultaneously revert key mechanisms of platinum resistance. Moreover, we define efflux pumps of the MRP family as secondary targets of DIPH and its derivatives. We provide a conclusive model proposing a pharmacological strategy of antidromically targeting CP transport mechanisms in Fig. 6.

## Conflict of interest

The authors declare no conflict of interest.

## Author contributions

JDK, JT, and TGO designed the experiments. MW, USW, DM, RAH, KM, BOG, and KE conducted the experiments and/or acquired data. PW, PB, TGO, DN, and HN provided clinical material and/or reagents. JT, JDK, USW, MM, DM, TGO, BOG, and SS analyzed the data. JDK, JT, USW, and MM wrote the manuscript and/or designed the figures. All authors read and approved the final version of the manuscript.

## Supporting information


**Fig. S1.** Structural formula of DIPH and its mehylated derivatives me‐DIPH and me_2_DIPH.
**Fig. S2.** Reduced systemic toxicity by co‐application of me_2_‐DIPH in C57BI/6 mice treated weekly with high dose CP as monotherapy or in combination with me_2_‐DIPH (10 mg/kg, 30 minutes before CP).
**Fig. S3.** DIPH and its derivatives augment DNA platination in A2780 cells across a broad range of CP concentrations.
**Fig. S4.** Null‐effect by co‐application of DIPH on CP‐induced DNA adduct formation in lung cancer cells and on tumor progression in a mouse model of KRAS‐mediated primary lung cancer.
**Fig. S5.** In vitro toxicity of DIPH+me‐DIPH in human tumor cell lines.
**Fig. S6.** The effect of DIPH on the anti‐tumor efficacy of long term CP treatment.
**Fig. S7.** The effect of DIPH on the anti‐tumor efficacy of short term CP treatment.
**Fig. S8.** Carboplatin‐sensitization by DIPH.
**Fig. S9.** Viability and caspase 3/7 kinetics in TOV‐21G ovarian cancer cell treated with DIPH and CP.
**Fig. S10.** Methylation of DIPH increases its CP‐sensitization effect.
**Fig. S11.** 3D superposition of similar compounds to DIPH.
**Fig. S12.** Detection of MRP2, MRP3 and MRP5 transcripts.
**Table S1.** The table lists all used cell lines, their medium conditions and CP sensitivity status.
**Table S2.** Effect of small molecules* on the accumulation of Pt‐(GpG) DNA adducts in critical target cells of CP treated mice.
**Table S3.** Prevalent augmentation of DNA platination (Pt‐GpG) in CP‐exposed human tumor cell lines by pre‐treatment with DIPH, me‐DIPH or me_2_‐DIPH.

## Data Availability

The manuscript contains all relevant data. The original set of raw data will be made available upon reasonable request.
