# Supplementary material for: Diphenhydramine increases the therapeutic window for platinum drugs by simultaneously sensitizing tumor cells and protecting normal cells
Source: Mol Oncol. 2020 Mar 10;14(4):686–703. doi: 10.1002/1878-0261.12648 (PMC7138396; doi:10.1002/1878-0261.12648)
Supplement: Supplementary file 1 — Fig. S1. Structural formula of DIPH and its mehylated derivatives me‐DIPH and me2DIPH. Fig. S2. Reduced systemic toxicity by co‐application of me2‐DIPH in C57BI/6 mice treated weekly with high dose CP as monotherapy or in combination with me2‐DIPH (10 mg/kg, 30 minutes before CP). Fig. S3. DIPH and its derivatives augment DNA platination in A2780 cells across a broad range of CP concentrations. Fig. S4. Null‐effect by co‐application of DIPH on CP‐induced DNA adduct formation in lung cancer cells and on tumor progression in a mouse model of KRAS‐mediated primary lung cancer. Fig. S5. In vitro toxicity of DIPH+me‐DIPH in human tumor cell lines. Fig. S6. The effect of DIPH on the anti‐tumor efficacy of long term CP treatment. Fig. S7. The effect of DIPH on the anti‐tumor efficacy of short term CP treatment. Fig. S8. Carboplatin‐sensitization by DIPH. Fig. S9. Viability and caspase 3/7 kinetics in TOV‐21G ovarian cancer cell treated with DIPH and CP. Fig. S10. Methylation of DIPH increases its CP‐sensitization effect. Fig. S11. 3D superposition of similar compounds to DIPH. Fig. S12. Detection of MRP2, MRP3 and MRP5 transcripts. Table S1. The table lists all used cell lines, their medium conditions and CP sensitivity status. Table S2. Effect of small molecules* on the accumulation of Pt‐(GpG) DNA adducts in critical target cells of CP treated mice. Table S3. Prevalent augmentation of DNA platination (Pt‐GpG) in CP‐exposed human tumor cell lines by pre‐treatment with DIPH, me‐DIPH or me2‐DIPH. [file MOL2-14-686-s001.pdf]

DIPH

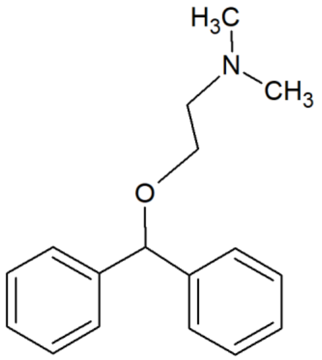

2-Diphenylmethoxy-N,N-dimethylethylamine (Diphenhydramine)

me-DIPH

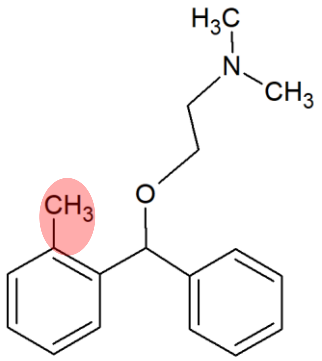

N,N-Dimethyl-2-(2-methyl-benzhydryloxy)ethylamine (Orphenadrine)

me_2-DIPH

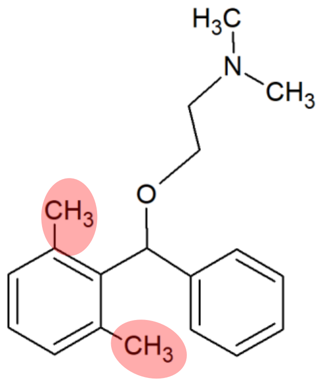

2-[(2,6-Dimethylphenyl) (Phenyl) Methoxy]-N,N-Dimethylethanamine

Supplementary Figure 1: Structural formula of DIPH and its mehylated derivatives me-DIPH and me_2DIPH. The Figure shows the lead compound DIPH and ist derivatives, which are methylated (marked in red) at position C2 or position C2+C6 of the phenylring, respectively.

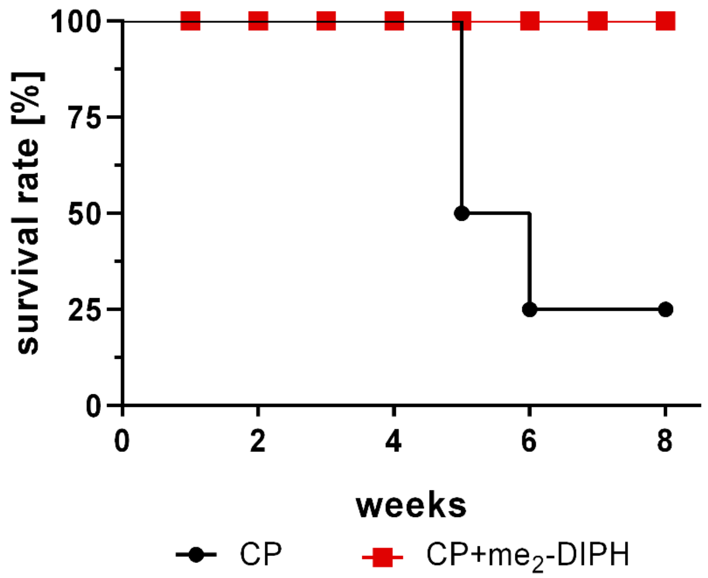

Supplementary Figure 2: Reduced systemic toxicity by co-application of me$_2$-DIPH in C57Bl/6 mice treated weekly with high dose CP as monotherapy or in combination with me$_2$-DIPH (10 mg/kg, 30 minutes before CP). Animals were observed for 8 weeks and were sacrificed when weight loss was >20%.

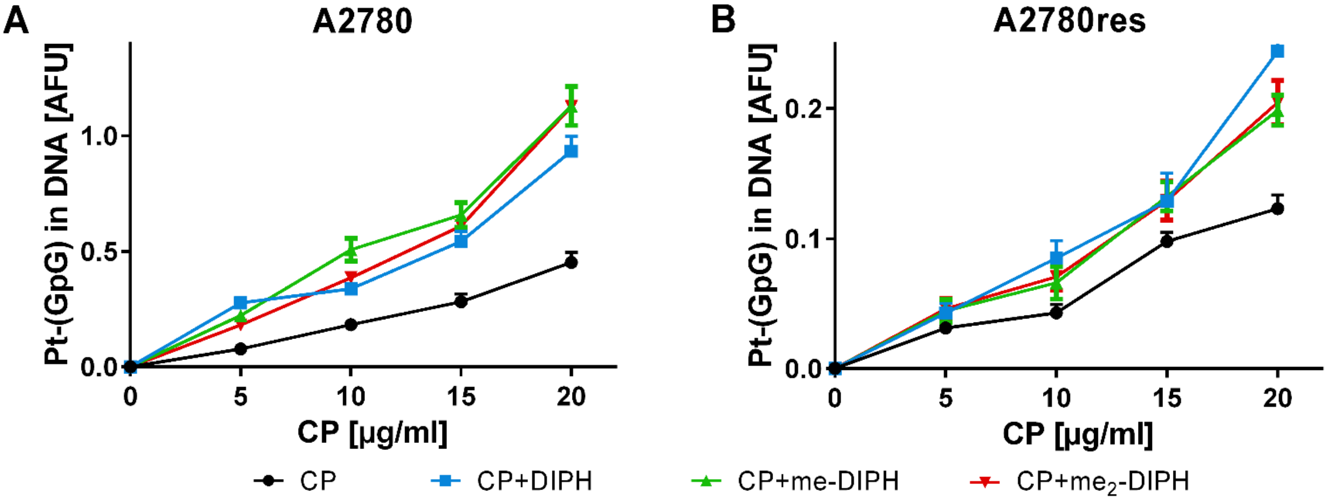

Supplementary Figure 3: DIPH and its derivatives augment DNA platination in A2780 cells across a broad range of CP concentrations. (**A**)CP-sensitive and (**B**) CP-resistant A2780 ovarian cancer cells were exposed to various concentrations of CP (5 - 20 µg/ml; 4h) alone or after pre-treatment with DIPH: (40 µg/ml), me-DIPH (20 µg/ml) or me_2-DIPH (10 µg/ml) 30 minutes before and throughout exposure. Cells were immuno-stained for Pt-(GpG) adducts and measured by ICA analysis as in Figure 2). Note different scales.

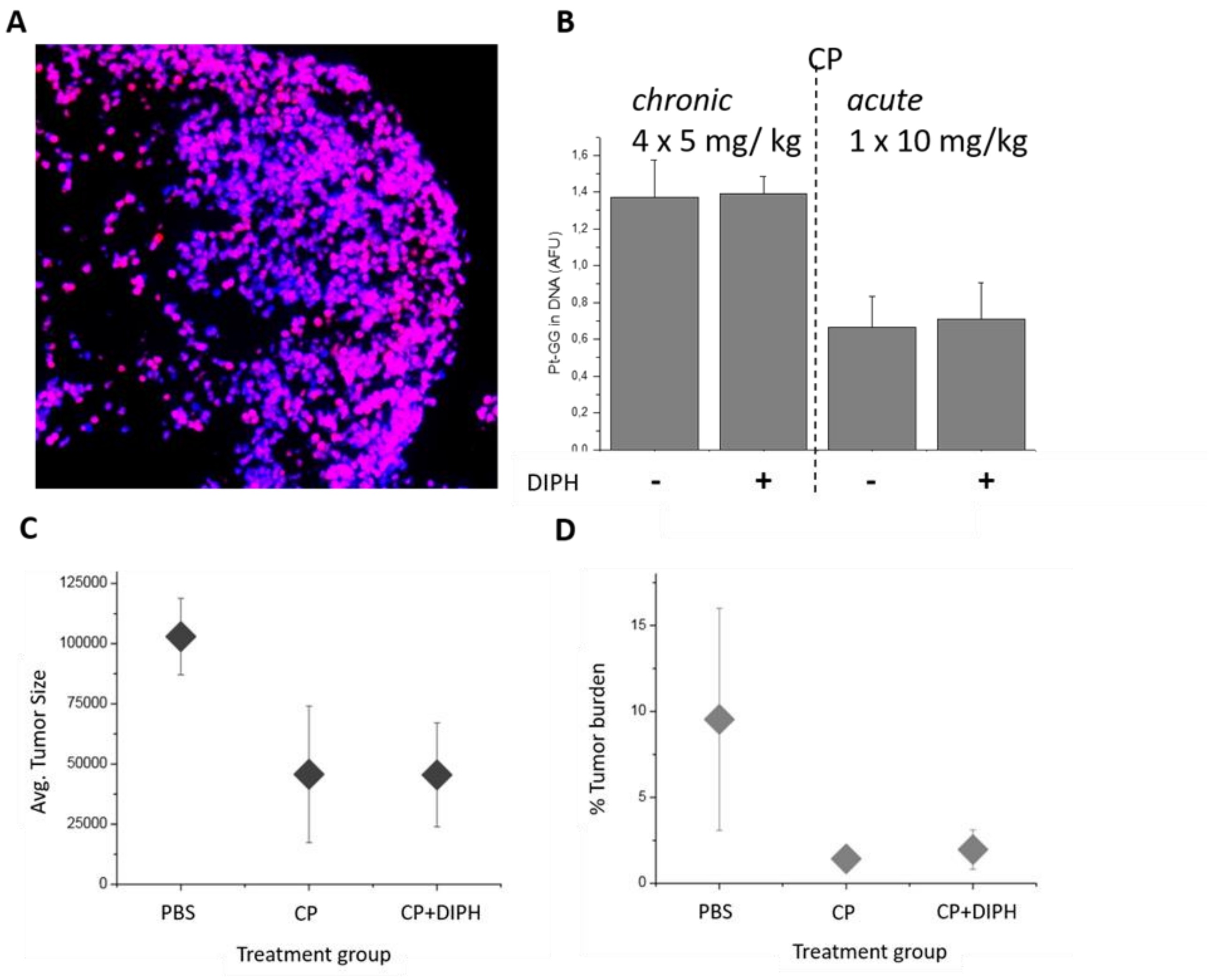

Supplementary Figure 4: Null-effect by co-application of DIPH on CP-induced DNA adduct formation in lung cancer cells and on tumor progression in a mouse model of KRAS-mediated primary lung cancer. (**A**) Visualization of Pt-(GpG) adducts (red) in the nuclear DNA (blue) of a lung cryo-section harboring a tumor nodule 24 h after a single dose of CP (10 mg/kg, i.p.). (**B**) DNA adduct levels in primary lung tumor cells of mice 24 h after single CP treatment (10 mg/kg) or repetitive treatments (4 x 5 mg/kg within 2 weeks) (**C**) Average tumor size and average tumor burden per lung section were determined by Bioquant image analysis software after H&E staining of tissue sections from mice treated with CP (7 mg/kg) once weekly for 4 weeks, or were additionally pre-treated with DIPH (57 mg/kg), or were mock-treated with PBS. For more experimental details see Oliver et al., 2011.

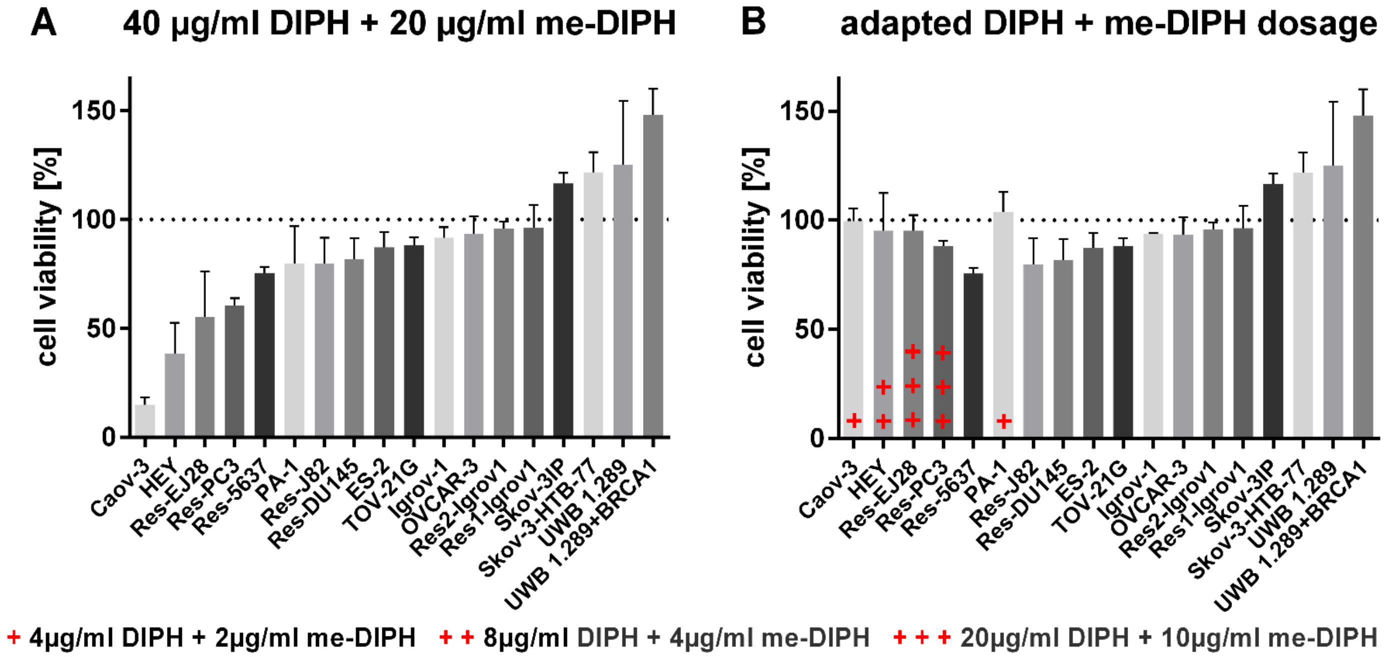

Supplementary Figure 5: In vitro toxicity of DIPH+me-DIPH in human tumor cell lines. (A) Cell viability of 18 different cancer cell lines treated with DIPH (40 µg/ml) and me-DIPH (20 µg/ml) for 52 h. **(B)** In order to reduce toxicity, the concentration of DIPH and me-DIPH was adapted in indicated cell lines. Normalized percentages of cell viability were averaged from three independent experiments and are reported as mean ± SD.

Supplementary Figure 6

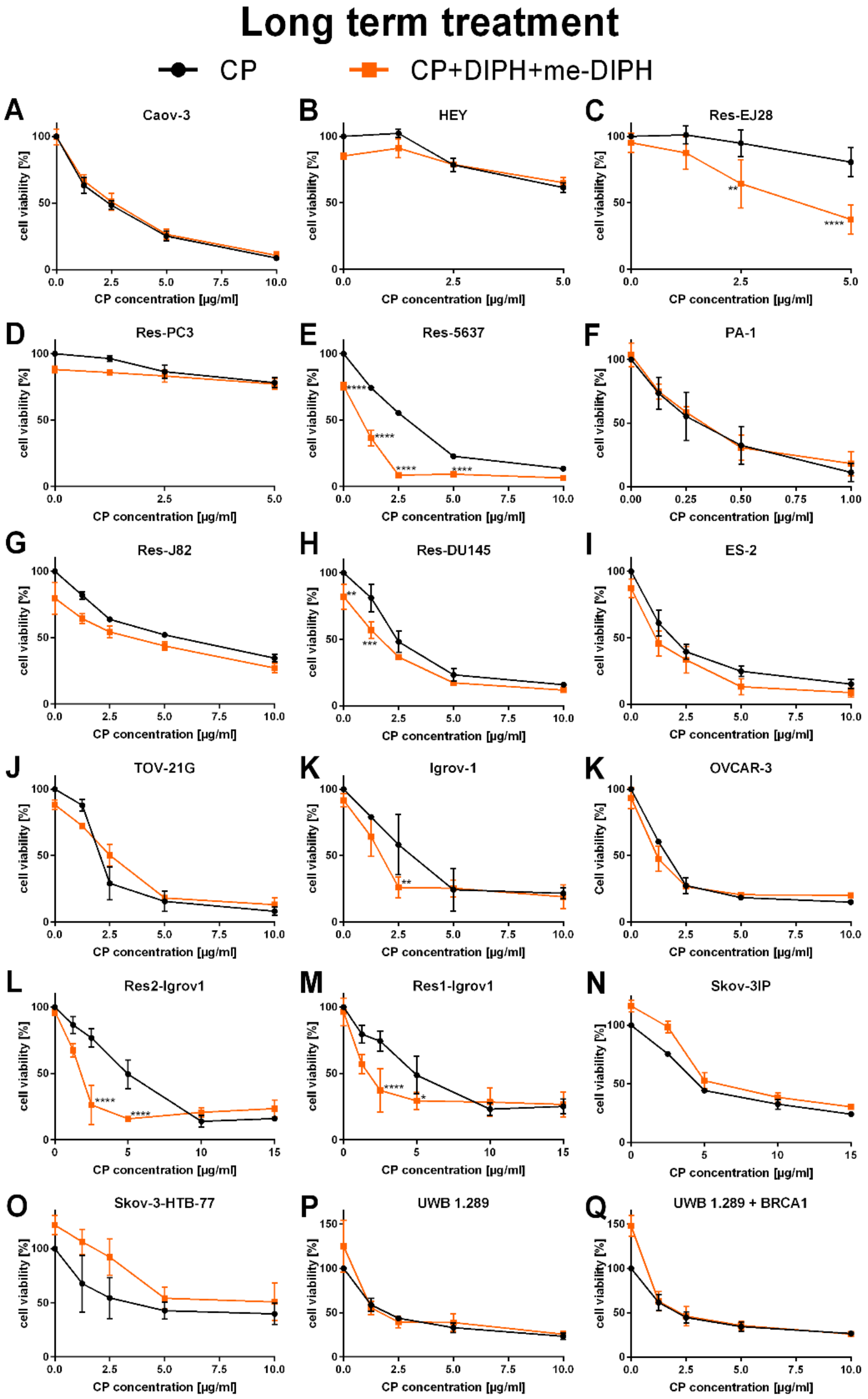

Supplementary Figure 6: The effect of DIPH on the anti-tumor efficacy of long term CP treatment. (A-Q) Viability dose-response curves following long term CP treatment for 48 h (black line) or pretreatment for 4 h with DIPH and me-DIPH (concentrations as indicated in Supplemental figure 2B) followed by combined treatment for 48 h (orange line). Normalized percentages of cell viability were averaged from three independent experiments and are reported as mean ± SD. For statistical analysis two-way ANOVA test ($p=0.05$) was used (*: $p \leq 0.05$; **: $p \leq 0.01$; ***: $p \leq 0.001$; ****: $p \leq 0.0001$).

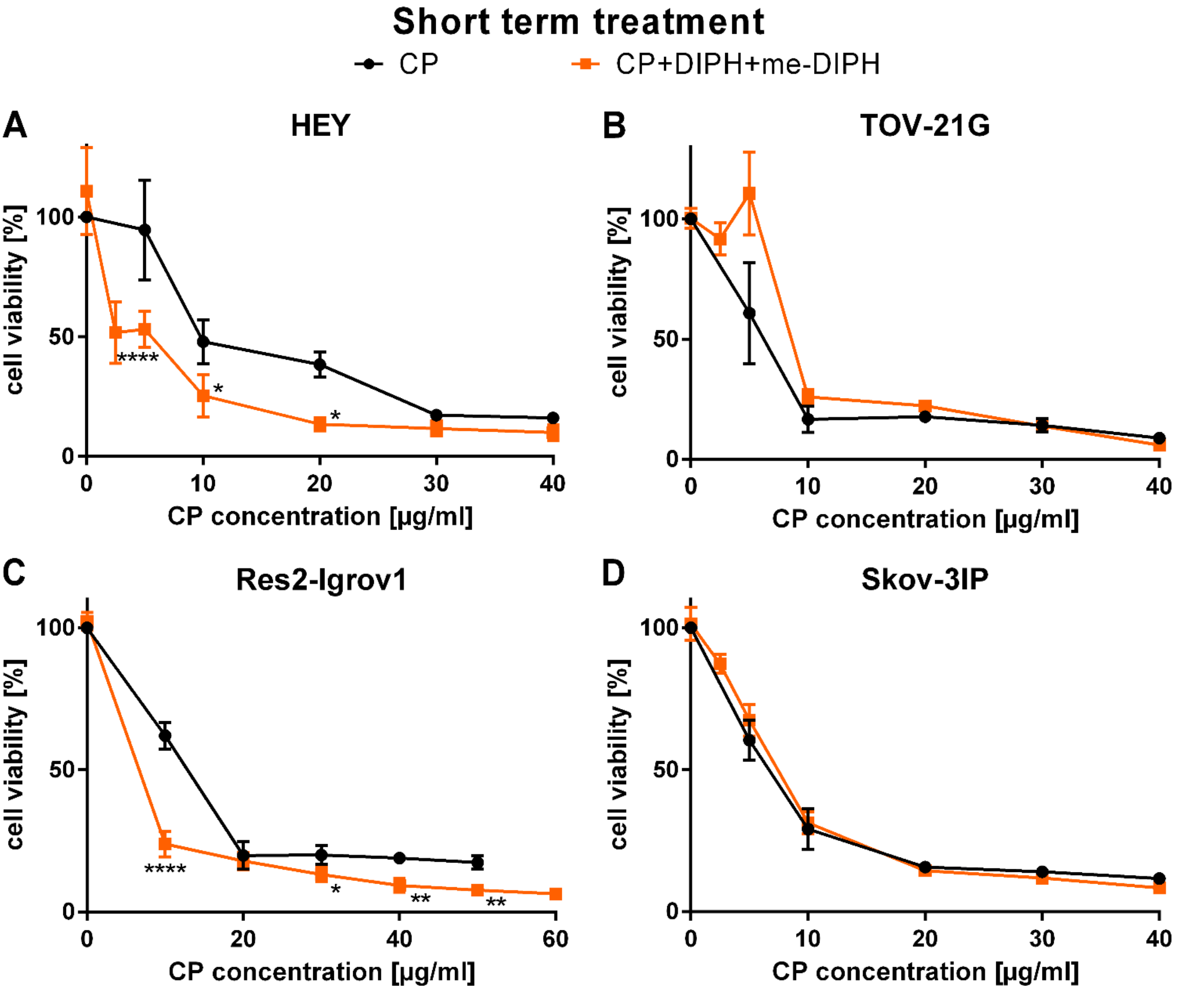

Supplementary Figure 7: The effect of DIPH on the anti-tumor efficacy of short term CP treatment. (A-D) Viability dose-response curves following short term CP treatment for 4 h (black line) or pretreatment for 1 h with DIPH (40 µg/ml) and me-DIPH (20 µg/ml) for 4 h (orange line). After 48 h the cell viability was measured. Normalized percentages of cell viability were averaged from three independent experiments and are reported as mean ± SD. For statistical analysis two-way ANOVA test ($p=0.05$) was used (*: $p \leq 0.05$; **: $p \leq 0.01$; ****: $p \leq 0.0001$).

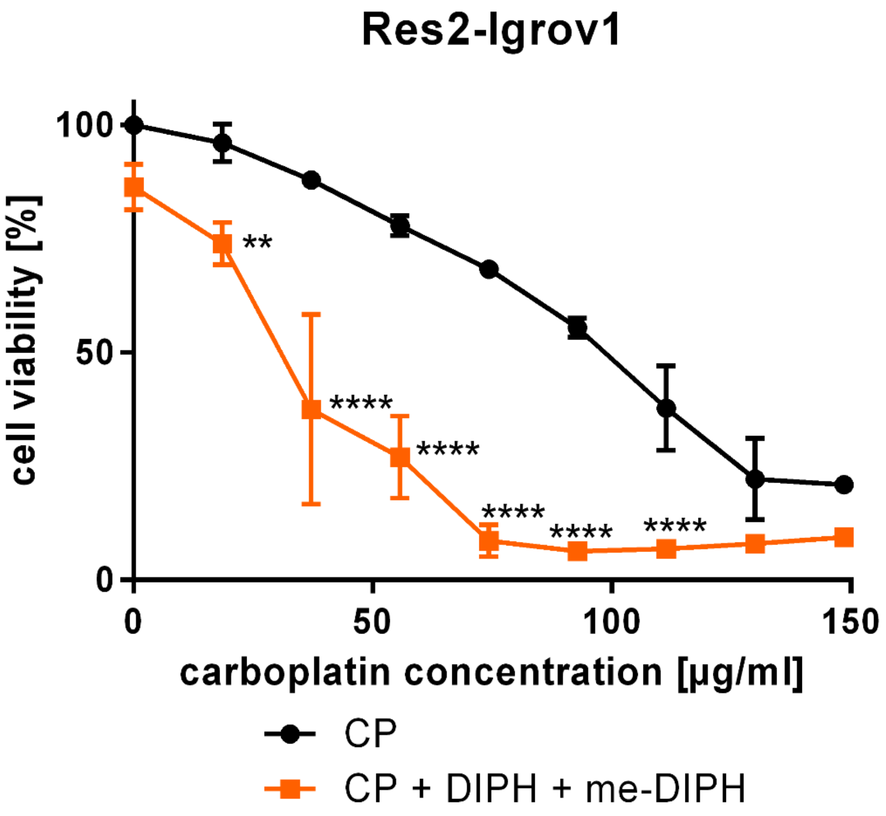

Supplementary Figure 8: Carboplatin-sensitization by DIPH. Viability dose-response curve in Res2-Igrov1 cells following carboplatin treatment (black line) or pretreatment for 4 h with DIPH (40 µg/ml) and me-DIPH (20 µg/ml) followed by combined treatment for 48 h. Normalized percentages of cell viability were averaged from three independent experiments and are reported as mean ± SD. For statistical analysis two-way ANOVA test ($p=0.05$) was used (**: $p \leq 0.01$; ****: $p \leq 0.0001$).

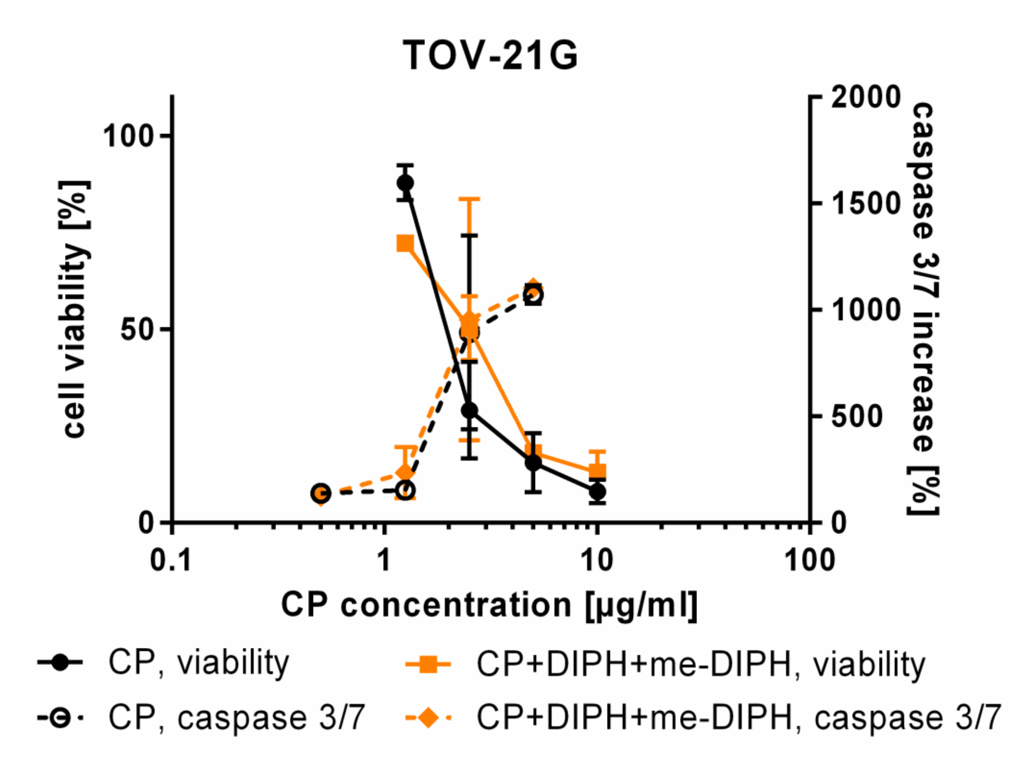

Supplementary Figure 9: Viability and caspase 3/7 kinetics in TOV-21G ovarian cancer cell treated with DIPH and CP. Viability dose-response curves (continuous line) and caspase 3/7 activity (dashed line) in TOV-21G ovarian cancer cells, following CP (black line) or CP with DIPH and me-DIPH treatment (orange line).

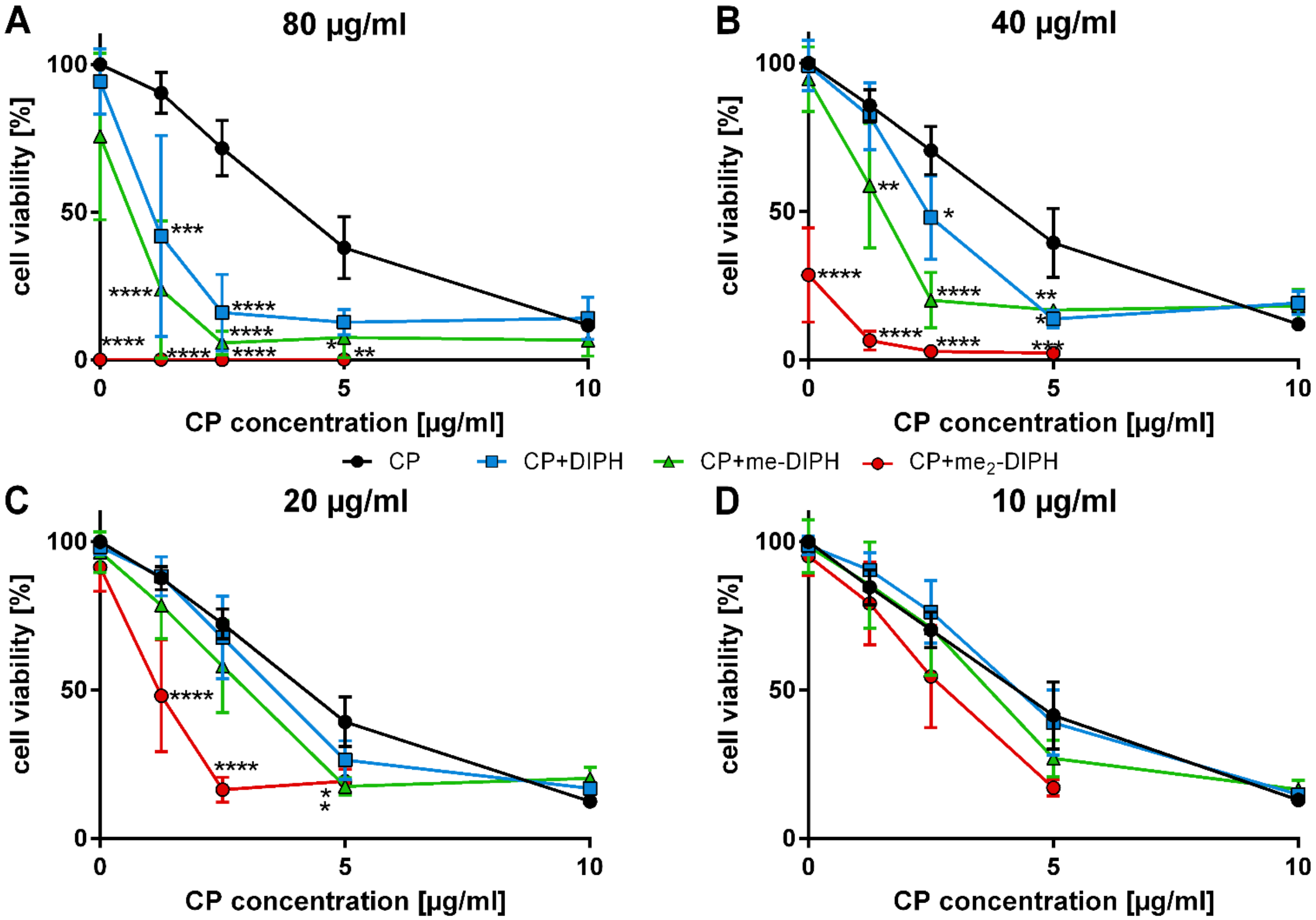

Supplementary Figure 10: Methylation of DIPH increases its CP-sensitization effect. Viability dose-response curves of platinum-resistant Res2-Igrov1 cells following pretreatment for 4 h with DIPH or its methylated derivatives followed by 48 h combined treatment with CP. Cells were treated with **(A)** 80 µg/ml, **(B)** 40 µg/ml, **(C)** 20 µg/ml or **(D)** 10 µg/ml DIPH or ist methylated derivatives me-DIPH or me_2-DIPH. Normalized percentages of cell viability were averaged from three independent experiments and are reported as mean ± SD. For statistical analysis two-way ANOVA test ($p=0.05$) was used (*: $p \le 0.05$; **: $p \le 0.01$; ***: $p \le 0.001$; ****: $p \le 0.0001$).

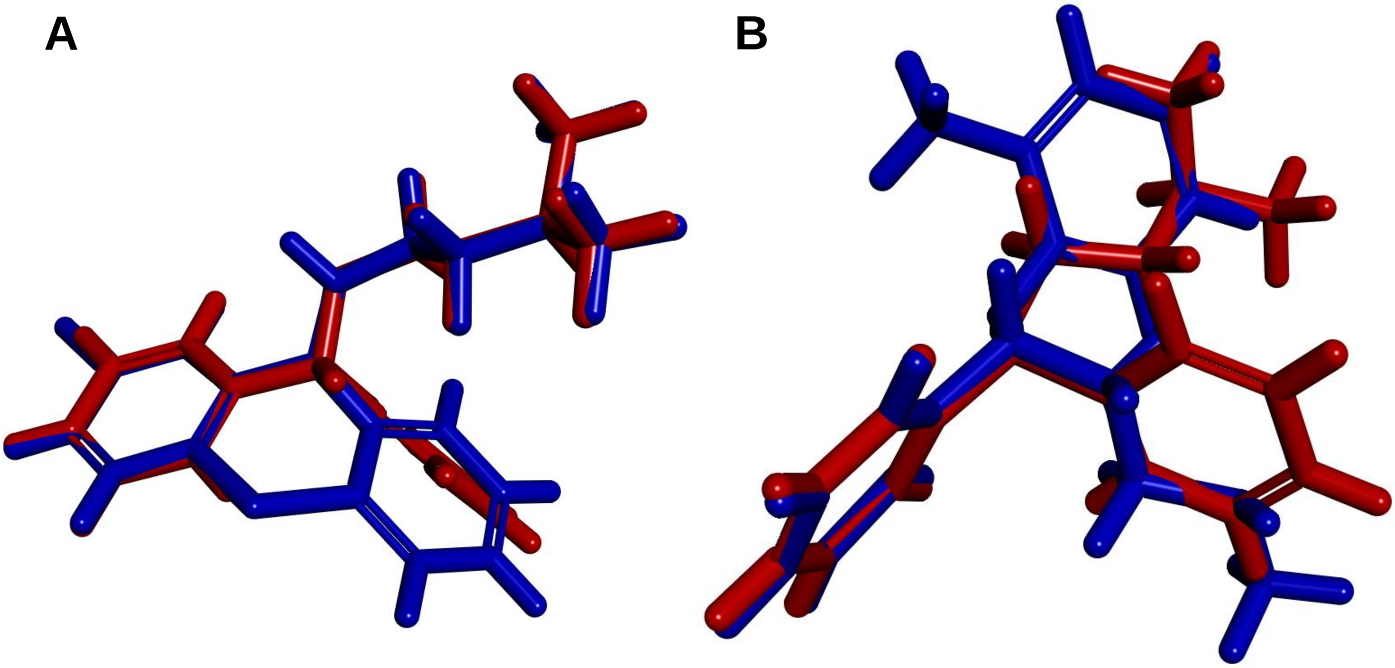

Supplementary Figure 11: 3D superposition of similar compounds to DIPH. By applying a combined structural similarity search approach to DIPH (red), two highly similar compounds could only be identified by 3D-similarity. (**A**) DIPH superposed with chloroprothixene, which shares a low 2D-similarity (structural) of 0.1 but a high 3D-similarity (dimensional) of 0.513 (RMSD-value) calculated by a superposition algorithm. (**B**) DIPH is superposed with atomoxetine (blue) for which a tanimoto similarity of 0.12 and a 3D-similarity (RMSD) of 0.655 was calculated.

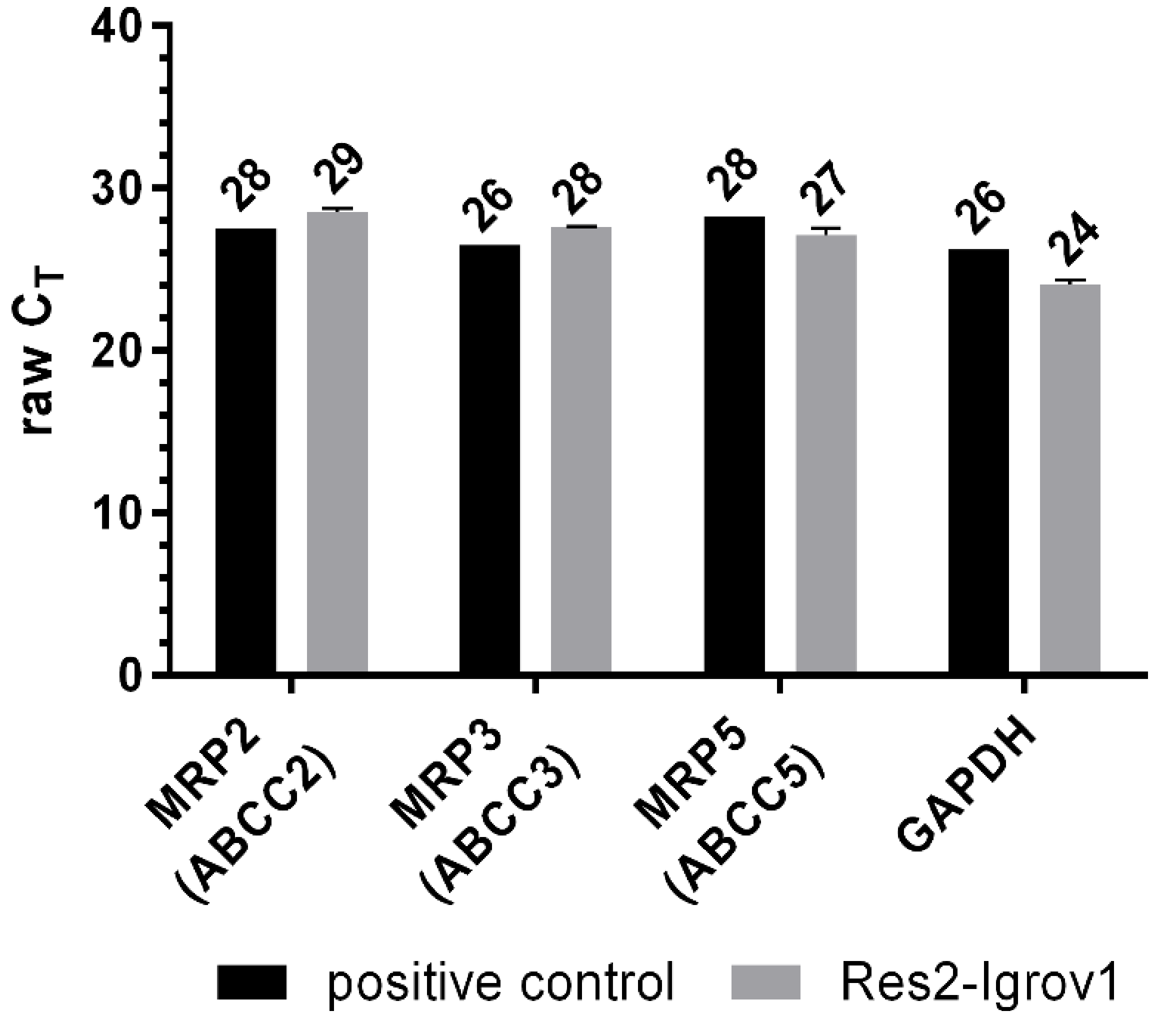

Supplementary Figure 12: Detection of MRP2, MRP3 and MRP5 transcripts. The bar chart shows the raw C_T of MRP2, MRP3 and MRP5 transcripts expression as measured in Res2-Igrov1 cells (grey) and in positive control Human XpressRef Universal Total RNA (black, both diluted 1:100).

Supplementary Table 1: The table lists all used cell lines, their medium conditions and CP sensitivity status.

Cell line	Characteristics	Cultured in	CP sensitivity status
**Igrov-1**	human ovarian cancer cell line derived from adenocarcinoma	RPMI 1640 supplemented with 2mM glutamine, 10 % fetal calf serum, 100 U/ml penicillin and 100 mg/ml streptomycin	sensitive
**A2780**	human ovarian cancer cell line	RPMI-1640 medium supplemented with 10 % FBS, 1 % penicillin and 1 % streptomycin	sensitive
**A2780res**	human ovarian cancer cell line	RPMI-1640 medium supplemented with 10 % FBS, 1 % penicillin and 1 % streptomycin	resistant
**Res1-Igrov1**	human ovarian cancer cell line derived from adenocarcinoma, *in vitro* developed platinum-resistance	RPMI 1640 supplemented with 2mM glutamine, 10 % fetal calf serum, 100 U/ml penicillin and 100 mg/ml streptomycin	resistant
**Res2-Igrov1**	human ovarian cancer cell line derived from adenocarcinoma, *in vitro* developed platinum-resistance	RPMI 1640 supplemented with 2mM glutamine, 10 % fetal calf serum, 100 U/ml penicillin and 100 mg/ml streptomycin	resistant
**PA-1**	cancer cells derived from human teratocarcinoma at the ovary	RPMI 1640 supplemented with 2mM glutamine, 10 % fetal calf serum, 100 U/ml penicillin, 100 mg/ml streptomycin and Insulin at 9 µg/ml	sensitive
**Skov-3IP**	platinum-resistant and highly malignant ovarian cancer cells which were originally obtained by in vivo passaging of Skov-3 cells in immunodeficient nude mice	DMEM high glucose, supplemented with 2mM glutamine, 10 % fetal calf serum, 100 U/ml penicillin, 100 mg/ml streptomycin, 1x MEM non-essential amino acid solution and 10 mM HEPES buffer	resistant
**UWB 1.289**	BRCA1-null human ovarian cancer cell line UWB1.289 from a tumor of papillary serous histology, UWB1.289 carries a germline BRCA1 mutation within exon 11 and has a deletion of the wild-type allele	1:1 mixture of RPMI-1640 and MEGM Bullet Kit, 10 % fetal calf serum, 100 U/ml penicillin and 100 mg/ml streptomycin	sensitive
**UWB1.289+BRCA1**	UWB1.289+BRCA1 is a stable cell line derived from UWB1.289 (ATCC CRL-2945), a BRCA1-null human ovarian cancer line, in which wild-type BRCA1 was restored	50% RPMI-1640 and 50% MEGM Bullet Kit, 10 % fetal calf serum, 100 U/ml penicillin, 100 mg/ml streptomycin and 200 µg/ml Geneticin/G-418	sensitive
**HEY**	human ovarian cancer cell line	DMEM supplemented with 2 mM L-Glutamine, 10 % fetal calf serum, 100 U/ml penicillin and 100 mg/ml streptomycin	resistant
**Skov-3-HTB-77**	derived from the ascitic fluid from a 64 year old caucasian female with an ovarian tumour	McCoys 5a medium supplemented L-glutamine, bicarbonate, 10% fetal calf serum, 100 U/ml penicillin and 100 mg/ml streptomycin	sensitive

**TOV-21G**	human ovarian adenocarcinoma cell line	1:1 mixture of MCDB 105 medium containing 1.5 g/L sodium bicarbonate and Medium 199 containing 2.2 g/L sodium bicarbonate, supplemented with 15 % fetal calf serum, 100 U/ml penicillin and 100 mg/ml streptomycin	sensitive
**ES-2**	ovarian carcinoma	McCoy’s 5a medium supplemented with 10 % fetal calf serum, 100 U/ml penicillin and 100 mg/ml streptomycin	sensitive
**Caov-3**	human ovarian adenocarcinoma,	DMEM high glucose supplemented with pyruvate, 10 % fetal calf serum, 100 U/ml penicillin and 100 mg/ml streptomycin	sensitive
**OVCAR-3**	human ovarian adenocarcinoma	RPMI 1640 supplemented with 2 mM glutamine, 0.01 mg/ml bovine insulin, 20 % fetal calf serum, 10 mM HEPES buffer, 100 U/ml penicillin and 100 mg/ml streptomycin	sensitive
**Res-PC3**	human prostate adenocarcinoma cells derived from bone metastasis, *in vitro* developed platinum-resistance	DMEM with 4.5 g/L glucose supplemented with 10 % fetal calf serum, 1x MEM non-essential amino acid solution and 10 mM HEPES buffer and 0.5 µg/ml cisplatin was freshly added to each passage	resistant
**Res-DU145**	human prostate cancer cells from brain metastases, *in vitro* developed platinum-resistance	DMEM with 4.5 g/L glucose supplemented with 10 % fetal calf serum, 1x MEM non-essential amino acid solution and 10 mM HEPES buffer and 0.5 µg/ml cisplatin was freshly added to each passage	resistant
**Res-5637**	human bladder cancer cells, *in vitro* developed platinum-resistance	RPMI 1640 supplemented with 10 % fetal calf serum and 0.5 µg/ml cisplatin was freshly added to each passage	resistant
**Res-EJ28**	human bladder cancer cells, *in vitro* developed platinum-resistance	DMEM with 4.5 g/L glucose supplemented with 10 % fetal calf serum, 1x MEM non-essential amino acid solution and 10 mM HEPES buffer and 2 µg/ml cisplatin was freshly added to each passage	resistant
**Res-J82**	human bladder cancer cells, *in vitro* developed platinum-resistance	DMEM with 4.5 g/L glucose supplemented with 10 % fetal calf serum, 1x MEM non-essential amino acid solution and 10 mM HEPES buffer and 0.25 µg/ml cisplatin was freshly added to each passage	resistant

Supplementary Table 2: Effect of small molecules* on the accumulation of Pt-(GpG) DNA adducts in critical target cells of CP treated mice

		Treatment* [mg/kg b.w.]	Kidney (proximal tubules)	Cochlea (marginal cells of stria vascularis)
			Mean adduct levels [% of CP only]	
CP	+	10	100	100
	Ranitidine	100	70	73
	Quinine	100	95	117
	Butylscopolamine	20	103	92
	Diphenhydramine	60	29	43
	Cimetidine	15	88	84
	Probenecis	150	100	115
	Desloratidine	240	97	107
	Fluoxetine	30	92	102
	Famotidine	20	105	122

Doses were adjusted to 50 % of the LD_{50} values reported in the literature.

* All drugs, tested herein, had previously been selected from literature survey and had been published in the subordinated context of CP-toxicity modulation or cellular CP-import/export.

Supplementary Table 3: Prevalent augmentation of DNA platination (Pt-GpG) in CP-exposed human tumor cell lines by pre-treatment with DIPH, me-DIPH or me_2-DIPH

Malignancy	Cell line	CP [20 µg/ml]		
		DIPH [40 µg/ml]	me-DIPH [20 µg/ml]	me_2-DIPH [10 µg/ml]
		Gain in mean adduct levels [% of CP only value for the cell line]		
Neuroblastoma	LAN-1	67	40	80
	LAN-1 CP-resistant	50	8	30
	Kelly	16	30	0
	Kelly CP-resistant	6	46	0
	Kelly Doxo-resistant	42	54	0
	Sy5y ($p53^{WT}$)	70	0	n.a.
Lung cancer (NSCLC)	A549	0	0	50
Testicular cancer	833K	45	57	0
	Susa	17	6	39
	GCT-27	0	12	n.a.
Breast cancer	MCF7	64	24	58
Prostate cancer	PC3	58	17	23
	RT112	25	32	19
	RT112 CP-resistant	24	34	60

Drug exposure of cells and DNA adduct analysis was as described in Figure 1, exposure: 3 h, pre-incubation: 30 min. n.a.: not analyzed
